# Development of Venous Thromboembolism Risk Prediction Models Based on Whole Blood Gene Expression Profiling Using 20 Machine Learning Algorithms: Comprehensive Analysis Study

**DOI:** 10.2196/75565

**Published:** 2026-01-16

**Authors:** Yedong Huang, Xiaoyun Chen, Guannan Bai, Yajun Zhao, Dapeng Kuang, Lin Zhang, Wei Lu

**Affiliations:** 1 Department of Radiation Oncology, Clinical Oncology School of Fujian Medical University, Fujian Cancer Hospital, Fuzhou, Fujian, China; 2 The School of Clinical Medicine, Fujian Medical University; Department of Respiratory and Critical Care Medicine, Fujian Provincial Geriatric Hospital, No. 147 Beihuan Middle Road, Fuzhou, China; 3 Children’s Hospital, Zhejiang University School of Medicine, Hangzhou, China; 4 Department of Health Management Centre, Zhongshan Hospital, Fudan University, Shanghai, China; 5 Department of Emergency and Critical Care, Shanghai General Hospital, Shanghai Jiao Tong University School of Medicine, Shanghai, China; 6 Department of Epidemiology and Preventive Medicine, The School of Public Health and Preventive Medicine, Monash University, Victoria, Australia; 7 Suzhou Industrial Park Monash Research Institute of Science and Technology, Monash University, Suzhou, China; 8 Department of Cardiovascular Surgery, The Quzhou Affiliated Hospital of Wenzhou Medical University, Quzhou People’s Hospital, Quzhou City, No.100 of Minjiang Road, Wenzhou, 325000, China, 86 13426423807, 86 13426423807

**Keywords:** venous thromboembolism, gene expression profiling, machine learning, biomarkers, predictive models, ROC, receiver operating characteristic curve, transcriptome

## Abstract

**Background:**

There is a lack of venous thromboembolism (VTE) risk prediction models based on gene expression information.

**Objective:**

This study aimed to construct a VTE prediction model based on whole blood gene expression profiling, by performing a comprehensive analysis of 20 machine learning (ML) algorithms.

**Methods:**

Two transcriptome datasets containing patients with VTE and healthy controls were obtained by searching the Gene Expression Omnibus database and used as the training and validation sets, respectively. Feature selection for model construction was performed on the training set using the least absolute shrinkage and selection operator and random forest, followed by the selection of the intersection of the chosen features. Subsequently, recursive feature elimination was applied to further refine the selected features. The selected features underwent model construction using 20 ML algorithms. The performance of the models was evaluated using various methods such as receiver operating characteristic and confusion matrix. The validation set was used for external model validation.

**Results:**

The final results demonstrated that all algorithm models, except for k-nearest neighbor, exhibited good performance in VTE prediction. External validation data indicated that 9 algorithm models had an area under the curve greater than 0.75. The confusion matrix analysis revealed that the algorithm models maintained high specificity in the external validation cohort.

**Conclusions:**

This study used 20 ML algorithms to construct VTE prediction models based on whole blood gene expression information, with 9 of these models demonstrating good diagnostic performance in external validation cohorts. The above models, when used in conjunction with D-dimer, may provide more valuable references for VTE diagnosis.

## Introduction

Venous thromboembolism (VTE) is a prevalent thrombotic disorder in clinical practice, consisting primarily of deep venous thrombosis and pulmonary embolism. VTE remains a major global health burden, affecting approximately 10 million people annually and ranking among the most common vascular diseases worldwide [1]. The pathogenesis of VTE is considered to be multifaceted and intricate, encompassing a multitude of factors, including but not limited to congenital genetic factors, malignancies, pregnancy in women, surgical trauma, and the use of oral contraceptives [2,3]. Despite the existence of relatively comprehensive prevention and assessment systems, the high mortality rate associated with VTE remains a significant concern among clinical practitioners. According to a study by Heit et al [4], the short-term (30-d) survival rate for deep vein thrombosis is approximately 94.5%, whereas the short-term survival rate for pulmonary embolism is only 66.8%.

In clinical practice, the diagnostic modalities most frequently used for VTE include D-dimer measurement, ultrasonography, and venography [5]. Among these methods, venography is considered the gold standard for diagnosing VTE. However, due to its invasive nature and the potential risk of contrast-induced renal impairment and allergic reactions, its clinical application is limited [6]. The limitations of ultrasonography include its subjectivity and difficulty in detecting deep venous thrombosis [7]. D-dimer testing is known to lack specificity, as elevated D-dimer levels may be caused by conditions such as infection, autoimmune diseases, pregnancy, and childbirth, leading to a higher likelihood of false positive results [8]. Therefore, it is crucial to construct a VTE risk prediction model with high performance.

With the advancement of science and technology, artificial intelligence (AI) has become a widely studied and highly regarded field in recent years, particularly in the medical domain. As one of the methods for achieving AI, machine learning (ML) algorithms have tremendous potential in medical research [9-11]. The aim of this study was to construct a VTE risk prediction model based on whole blood gene expression information, using a comprehensive analysis of 20 ML algorithms and external validation, with the goal of providing a reference for clinical decision-making and VTE prevention. These models are not only designed to differentiate between existing VTE cases and controls, but also intended to detect high-risk patients in early or asymptomatic stages, thereby serving as predictive tools that may assist in clinical decision-making before definitive diagnostic imaging is performed.

## Methods

### Data and Source

The gene expression data of patients with VTE and healthy controls were retrieved and downloaded from the Gene Expression Omnibus (GEO) database and used as the training and validation groups, respectively, in this study. The GSE19151 dataset was used as the training set, while the GSE48000 dataset served as the validation set. Both datasets were generated using the Affymetrix Human Genome U133 Plus 2.0 microarray platform (GPL570). Table 1 shows the basic information of the 2 datasets, GSE19151 and GSE48000. The GSE19151 dataset comprises gene expression data from 70 patients with VTE and 63 healthy controls [12]; the GSE48000 dataset contains gene expression data from 107 patients with VTE and 25 healthy controls [13]. Data analysis was performed using the *Scikit-learn* (sklearn) module in Python (version 3.6; Python Software Foundation).

**Table 1.** Details of the Gene Expression Omnibus (GEO)^a^ datasets used in this study.

Dataset	GEO ID	Sample size (n)	PubMed ID	Number of genes	Country
Training set	GSE19151	133	21737128	13,515	USA
Validation set	GSE48000	132	25684211	31,412	USA

a GEO: Gene Expression Omnibus.

### Feature Selection

Least absolute shrinkage and selection operator (LASSO) regression analysis method was developed by Robert Tibshirani [14]. The principle of this method is to construct a penalty function to achieve the goal of shrinking regression coefficients and refining the model. While the principle of feature selection using random forest (RF) is mainly based on evaluating the contribution of each feature in the RF on each tree. In this study, we used the mean decrease in Gini impurity to measure feature importance in the RF model. The above 2 methods were used to select modeling features in the training set, and the intersection analysis was subsequently performed to reveal the features selected by both methods. This intersection strategy was designed to combine the strengths of both linear (LASSO) and nonlinear (RF) approaches and to reduce potential selection bias by focusing on consistently selected features. Although this intersection approach may sacrifice sensitivity, it enhances stability and reduces overfitting risk in high-dimensional settings. All feature selection procedures were performed exclusively on the training dataset to prevent information leakage from the validation data. The results were then visualized by a Venn plot.

### Recursive Feature Elimination for Model Simplification

The main idea of the recursive feature elimination (RFE) method is to repeatedly construct models and remove the most significant features, ranking the features according to the order in which they are eliminated. To achieve a more streamlined model without compromising performance, the features selected by the aforementioned method were further screened and simplified using the RFE method in our study.

### Construction of a VTE Risk Prediction Model Using 20 ML Algorithms

After completing the feature selection process, this study constructed the model using commonly used ML algorithms in the field, which included the following: adaptive boosting, artificial neural network, bagging, Bayesian ridge, decision tree, elastic net, extra tree (extremely randomized trees), gradient boosting, k-nearest neighbors (KNN) algorithm, LASSO, linear LASSO, linear regression, logistic regression, naïve Bayes, RF, ridge regression, ridge cross-validation (ridge regression with cross-validation), stochastic gradient descent, support vector machine, and voting (voting classifier). The algorithm code is taken from the sklearn website [15]. All models were constructed using 5-fold cross-validation.

### External Validation of the Model

In this study, GSE48000 was used as the external validation cohort to evaluate the generalization ability of the 20 ML models, ensuring the reproducibility of the model. The performance of the external validation was evaluated by the receiver operating characteristic (ROC) curve. All feature selection and model training were exclusively performed on the training dataset. The final trained models were then directly applied to the validation dataset without any retraining or adjustment, thereby ensuring a true external validation.

### Decision Curve Analysis

To evaluate the potential clinical use of the constructed models, we performed decision curve analysis (DCA) across both internal and external evaluation settings. The net benefit for each model was calculated using the standard formula:


$$NetBenefit=\frac{TP}{N}-\frac{FP}{N}*\frac{PT}{1-PT}$$

where TP and FP denote the numbers of true and false positives at a given threshold probability, and *n* is the total number of samples. DCA was conducted over a range of clinically relevant threshold probabilities from 0.05 to 0.5. The decision curves were generated using Python with appropriate packages compatible with sklearn.

### Ethical Considerations

This study involved secondary analysis of publicly available, deidentified transcriptomic datasets obtained from the GEO database. As per institutional and GEO data usage policies, such analysis does not require ethical approval or informed consent. For more information, please refer to the GEO data policy [16].

## Results

### The Features Selected by LASSO Regression and RF

To determine the optimal regularization strength in LASSO regression, we used 5-fold cross-validation to identify the λ value corresponding to the “one standard error rule” (λ₁se), which balances model simplicity and predictive performance. The regularization path was explored using the LASSO cross-validation function in sklearn, which automatically tests a logarithmic range of λ values. We selected 24 features corresponding to λ=−3.38.

**Figure 1.**
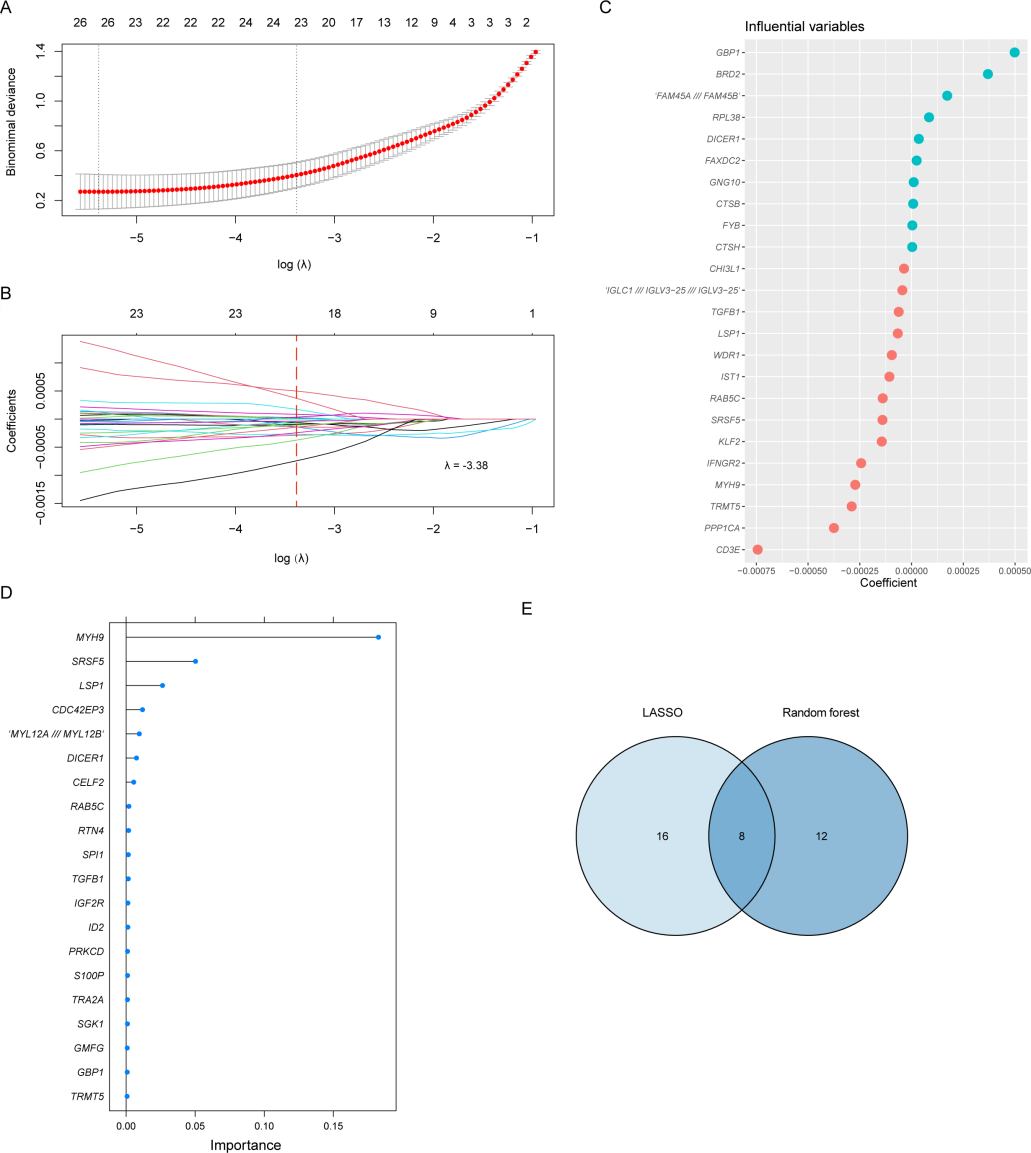
The process of selecting modeling features using least absolute shrinkage and selection operator (LASSO) and random forest methods.

The process of feature selection using LASSO regression is illustrated in Figure 1A and B and Figure 1C illustrates the 24 features selected by LASSO regression and their corresponding coefficients. The screening results of the RF are shown in Figure 1D, where the lollipop plot displays the 20 features selected by RF, with the length of the horizontal axis representing the corresponding importance of each gene. The Venn diagram in Figure 1E shows the intersection of the features selected by LASSO regression and RF methods. The 8 features selected by the intersection analysis are as follows: *TRMT5, TGFB1, SRSF5, RAB5C, MYH9, LSP1, GBP1,* and *DICER1*.

### Recursive Feature Elimination to Simplify the Model

**Figure 2.**
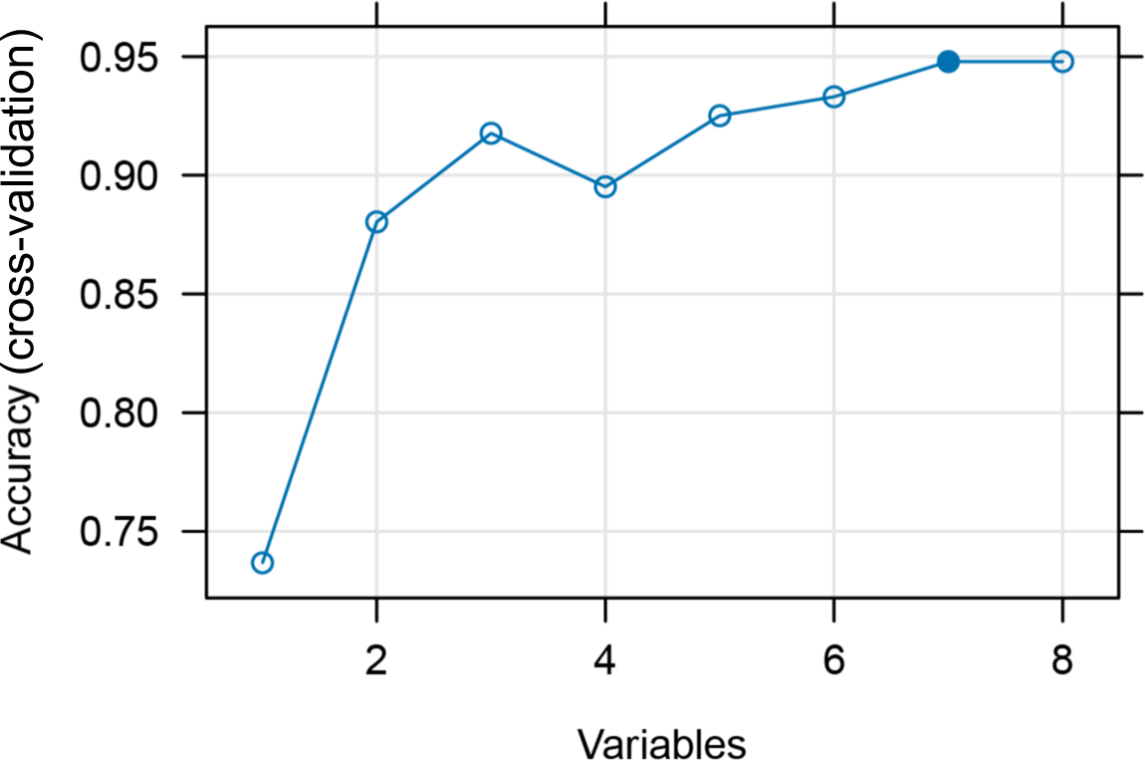
The process of simplifying modeling features using recursive feature elimination.

The above 8 features were further simplified using the RFE algorithm, which reduces costs and improves the generalizability of the model while removing redundant features. Figure 2 displays the results of the RFE calculations using a line graph. The results indicate that the accuracy of the model in cross-validation no longer significantly changes when the number of features is reduced to 7. The features further refined by the RFE algorithm include 7 genes: *SRSF5*, *MYH9*, *LSP1*, *RAB5C*, *TGFB1*, *DICER1*, and *GBP1*.

### Construction of VTE Risk Prediction Models Using 20 ML Algorithms

**Figure 3.**
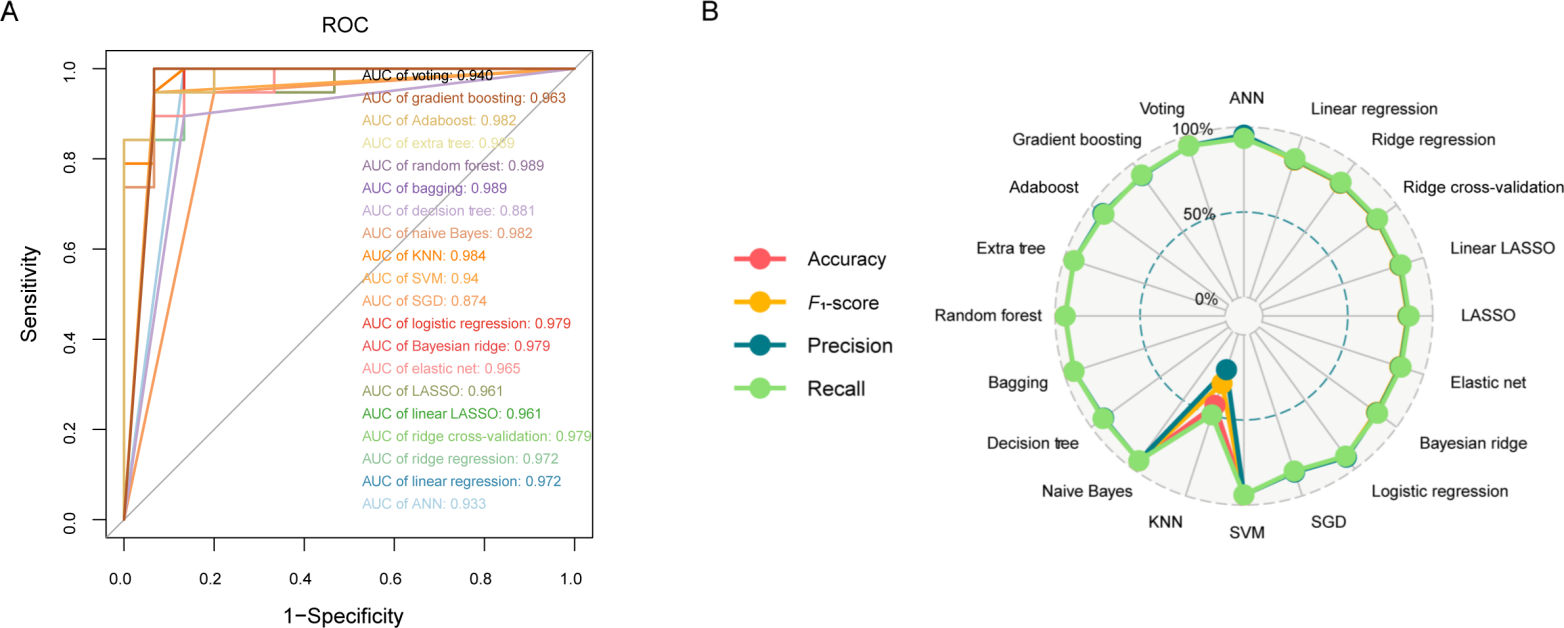
Performance comparison of 20 machine learning models using receiver operating characteristic (ROC) curves and radar plots. (**A**) ROC curves and area under the curve values based on 20 machine learning models. (**B**) Radar plots comparing accuracy, *F*_1_-score, precision, and recall across models. AdaBoost: adaptive boosting; ANN: artificial neural network; KNN: k-nearest neighbor; LASSO: least absolute shrinkage and selection operator; SGD: stochastic gradient descent; SVM: support vector machine.

Based on the 7 features selected, our study used 20 commonly used ML algorithms to construct the models and used 5-fold cross-validation to improve the accuracy of model evaluation and reduce random error. Figure 3A displays the ROC curves and corresponding area under the curve (AUC) values of the 20 ML models using different colored lines. Figure 3B depicts a radar chart showcasing the accuracy, *F*_1_-score, precision, and recall for 20 ML algorithms. The accuracy reflects the proportion of correctly predicted samples; precision reflects the proportion of actual VTE cases among samples classified as VTE by the model; recall represents the proportion of VTE cases correctly classified by the model among all VTE samples; the *F*_1_-score is a value used to comprehensively evaluate both recall and precision. As illustrated in Figure 3, most models exhibit an AUC value greater than 0.9, and the radar chart based on 4 parameters evaluation indicates that, except for the KNN model, all other ML models can make relatively accurate predictions for VTE.

**Figure 4.**
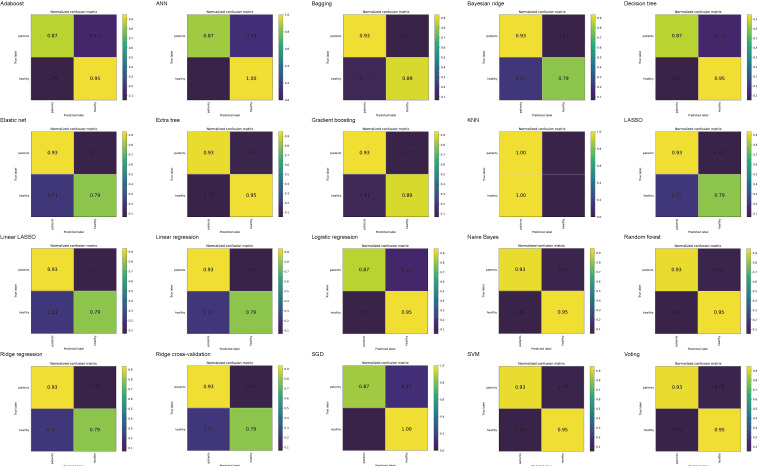
Confusion matrices for 20 machine learning algorithms. The top-left cell indicates the true positive, top-right indicates the false negative, bottom-left indicates the false positive, and bottom-right indicates the true negative. AdaBoost: adaptive boosting; ANN: artificial neural network; KNN: k-nearest neighbor; LASSO: least absolute shrinkage and selection operator; SGD: stochastic gradient descent; SVM: support vector machine.

Figure 4 presents the confusion matrices for 20 ML algorithms, among which the sensitivity of all models except for the KNN fluctuated between 87% and 93%, and the specificity fluctuated between 79% and 100%. The findings above indicate that, with the exception of the KNN model, the remaining 19 ML models possess favorable predictive efficacy for VTE. Multimedia Appendix 1 presents the DCA for 20 ML algorithms.

### External Validation of the Models

**Figure 5.**
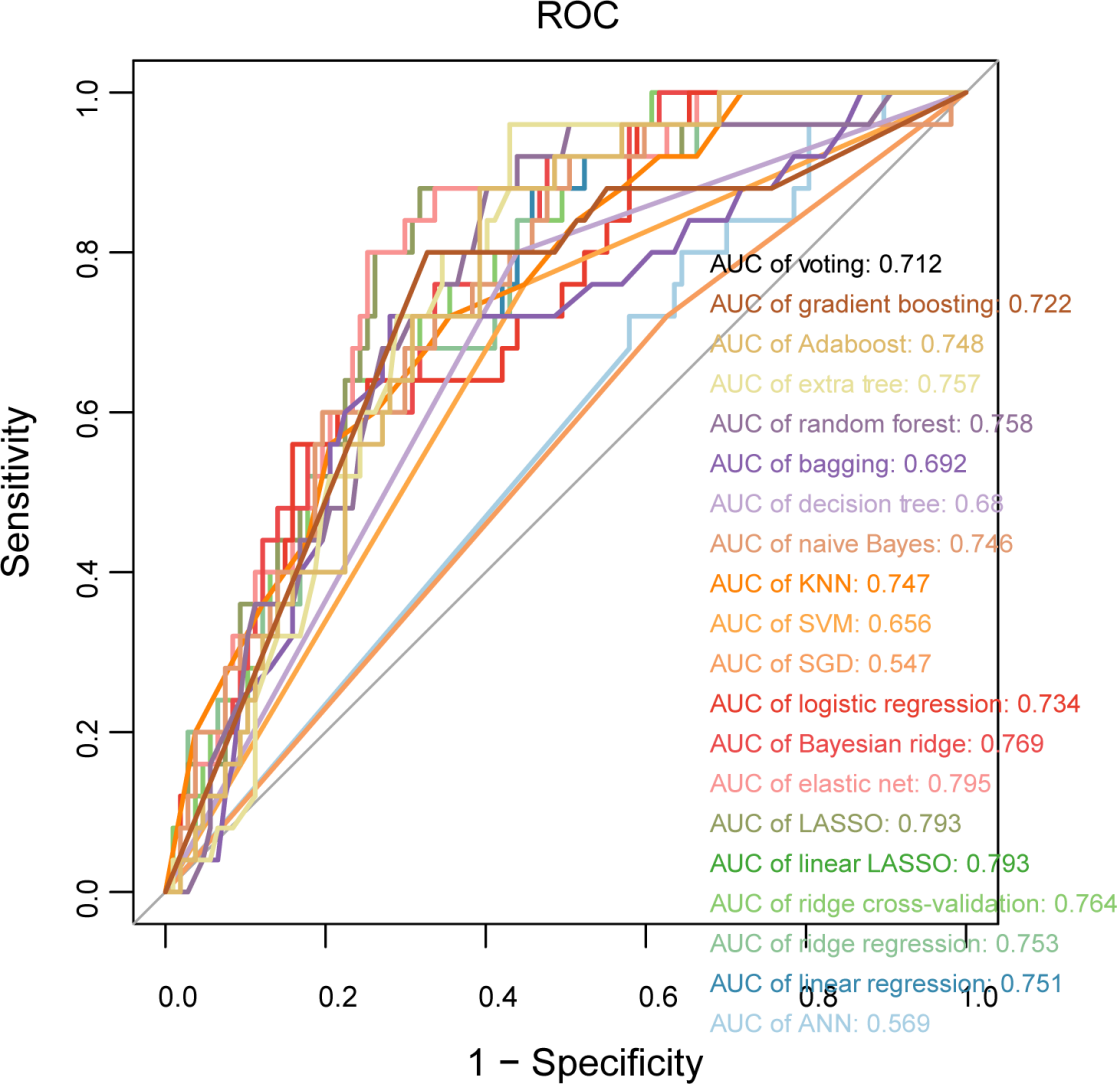
The external validation receiver operating characteristic (ROC) curve plot for the 20 machine learning models. AdaBoost: adaptive boosting; ANN: artificial neural network; KNN: k-nearest neighbor; LASSO: least absolute shrinkage and selection operator; SGD: stochastic gradient descent; SVM: support vector machine.

To ensure the reproducibility and generalizability of the model, this study performed external validation of the 20 ML models using the GSE48000 dataset. Figure 5 illustrates the ROC curves and the corresponding AUC based on the external validation dataset. The results revealed a significant decrease in the AUC of all models during external validation, but the AUC of the following 9 models remained above 0.75: extra tree, RF, Bayesian ridge, elastic net, LASSO, linear LASSO, ridge cross-validation, ridge regression, and linear regression. This result indicates that the predictive performance of the models corresponding to the 9 ML algorithms is relatively stable, and they exhibit strong generalizability.

**Figure 6.**
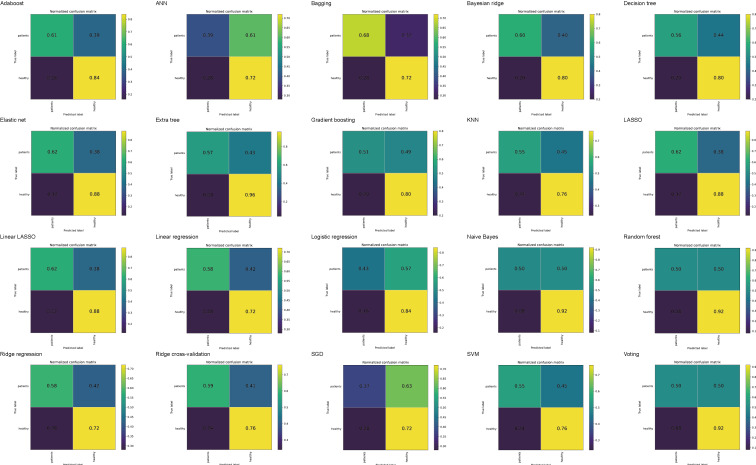
Confusion matrices of 20 machine learning algorithms during external validation. The top-left cell indicates the true positive, top-right indicates the false negative, bottom-left indicates the false positive, and bottom-right indicates the true negative. AdaBoost: adaptive boosting; ANN: artificial neural network; KNN: k-nearest neighbor; LASSO: least absolute shrinkage and selection operator; SGD: stochastic gradient descent; SVM: support vector machine.

Figure 6 displays the confusion matrices of 20 ML algorithms during external validation. The sensitivity of all models decreased significantly, ranging from 37% to 62%, whereas the specificity remained relatively stable, fluctuating between 72% and 96%. These findings suggest that the models still possess high specificity in the external validation dataset. Multimedia Appendix 2 displays the DCA for 20 ML algorithms in external validation.

## Discussion

### Principal Findings

As a rapidly advancing field in computer science, AI has the ability to process vast and intricate medical data for the diagnosis and prediction of potential clinical outcomes [17]. ML, as a component of AI, can extract valuable information and patterns from vast amounts of data through learning and analysis, thereby achieving AI [18]. In the medical field, ML is predominantly used for medical image analysis, disease prediction and risk assessment, health care management, decision support, and drug development, among other applications [19-21]. As the first study to construct a VTE risk prediction model based on whole blood gene expression information, the present research aims to empower the diagnosis of VTE through the use of ML algorithms. It is important to note that the current model was trained and validated only on healthy controls versus patients with VTE and does not assess its discriminative ability in populations with other inflammatory or thrombotic conditions. On the other hand, the external validation results indicate that the models developed in this study are characterized by low sensitivity and high specificity. According to the “No free lunch” theory proposed by Wolpert et al [22], a perfect model does not exist. In other words, if a certain ML model outperforms other algorithms in a particular evaluation metric, it must inevitably sacrifice some performance in other metrics as a trade-off. As a result of this constraint, it is not feasible to determine a flawless ML model as the conclusive outcome. Rather, a more fitting model is chosen by taking into account a comprehensive evaluation of diverse metrics.

At present, the models used for predicting the risk of VTE occurrence are predominantly built upon clinical features. Darzi et al [23] conducted a meta-analysis that included 17 studies, which indicated that VTE may be associated with the following factors: advanced age, elevated C-reactive protein levels, increased D-dimer and fibrinogen levels, tachycardia, thrombocytosis, leukocytosis, fever, leg swelling, malignancy, immobility, infection, and so on. In 2021, Pandor et al [24] conducted a systematic review of 24 VTE risk assessment models to evaluate their predictive performance. The results showed that the *C*-statistic was generally less than 0.7 for all models, with only a few models having favorable *C*-statistics (>0.8). Among these models, sensitivity fluctuated between 12% and 100%, while specificity varied between 7.2% and 100%. Based on the aforementioned findings, the study suggests that the existing VTE risk prediction models have generally weak predictive ability and high heterogeneity, and thus, does not recommend the use of any specific prediction model. Our study is an exciting and innovative attempt to discover modeling features based on gene expression analysis. In the internal validation, most of the models developed in this study exhibited good performance (AUC >0.90), while in the external validation, the data showed that 9 models had an AUC greater than 0.75. Considering clinical applicability, we note that models such as LASSO, ridge regression, and linear regression not only performed well in external validation (AUC >0.75), but also offer the advantages of simplicity, computational efficiency, and interpretability. In contrast, while ensemble models such as RF and extra trees demonstrated strong predictive power, their black-box nature may pose challenges for clinical interpretation. As such, we believe interpretable models like LASSO may be better suited as candidates for further clinical translation.

The current clinical diagnostic techniques for VTE primarily consist of D-dimer testing, ultrasonography, and venography [5]. Each of the aforementioned 3 diagnostic methods has their own limitations to varying degrees. In 2016, Crawford et al [25] conducted a meta-analysis of 1585 patients from 4 studies and reported that the sensitivity and specificity of D-dimer for diagnosing VTE were 80% to 100% and 23% to 63%, respectively. Although the sensitivity and specificity of D-dimer for diagnosing VTE may vary slightly across different studies, they generally exhibit high sensitivity and low specificity. The main reason for this phenomenon is that various conditions, including trauma, malignancy, and inflammation, can cause an elevation in D-dimer levels, leading to high sensitivity but low specificity in diagnosing VTE using D-dimer testing [26]. In summary, an isolated increase in D-dimer levels is insufficient to establish a diagnosis of VTE. The VTE risk prediction model developed in this study complements D-dimer well in terms of diagnostic performance. Moreover, since both tests use blood samples, they are highly compatible with each other in combination. The high specificity of the developed models, when used in combination with D-dimer testing, can assist clinicians in excluding false-positive cases that may be misdiagnosed as VTE by D-dimer alone. Furthermore, the joint use of the models and D-dimer has the potential to improve the diagnostic accuracy of VTE in patients. This patient population should be regarded as a priority for VTE prevention by clinicians to optimize VTE prevention measures, avoid wastage of medical resources, and achieve greater precision in VTE prevention. A potential clinical application of the gene expression-based models could be in a 2-step diagnostic pathway. For instance, the models may be used as a secondary triage tool following a positive D-dimer result to improve specificity and reduce unnecessary imaging procedures. Alternatively, they could be selectively applied to high-risk subpopulations (eg, older patients, postoperative individuals, or those with cancer) where D-dimer alone may be insufficiently specific. On the other hand, despite encouraging AUC values and specificity in external validation, we acknowledge that the observed sensitivity levels (ranging from 37%-62%) limit the standalone diagnostic capability of the models. This performance pattern may partly result from the significant class imbalance in the validation dataset and potential dataset shift. Importantly, the models are not intended to replace established diagnostic tools, but rather to serve as adjunctive decision aids. Their high specificity may be particularly valuable in ruling out false positives among D-dimer positive patients. Future work may consider ensemble approaches, risk stratification frameworks, or combining these models with clinical parameters to improve sensitivity while preserving specificity.

This study has several limitations. First, the modeling and external validation data were obtained from 2 sequencing cohorts in the GEO database (n=133; n=132), which may be considered a relatively small sample size, and the external validation set also exhibited a notable class imbalance (107 VTE vs 25 controls), which may have contributed to the observed reduction in sensitivity. Second, as the model has not yet been implemented in clinical practice, and both datasets were derived from US populations without detailed ethnicity annotations in the GEO records, its predictive performance in real-world clinical settings and across diverse ethnic backgrounds remains unverified. Third, we used a sequential feature selection strategy combining LASSO, RF, and RFE, aiming to enhance robustness and reduce dimensionality. However, we acknowledge that this approach may introduce complexity and the potential risk of overfitting, particularly when applied to algorithms that already incorporate internal regularization mechanisms. This redundancy could limit generalizability. Moreover, although some of the best-performing models in this study are inherently nontransparent, several interpretable models (eg, LASSO and logistic regression) were also included to ensure interpretability and practical applicability. In future work, we aim to further improve the explainability of complex models by using Shapley Additive Explanations or Local Interpretable Model-Agnostic Explanations–based interpretation frameworks, thereby enhancing their clinical interpretability and reliability. Future studies may benefit from alternative feature selection strategies, such as embedded or end-to-end feature learning methods within specific model frameworks, and from incorporating formal calibration analyses to improve clinical interpretability and reliability.

### Conclusions

The current study used ML algorithms to construct 20 VTE risk prediction models based on whole blood gene expression information. Notably, 9 of these models displayed favorable diagnostic performance in the external validation dataset. Thus, these models, in conjunction with D-dimer, have the potential to serve as a valuable reference for the clinical diagnosis of VTE.

## Supplementary material

10.2196/75565 Multimedia Appendix 1 Decision curve analysis (DCA) for 20 machine learning algorithms.

10.2196/75565 Multimedia Appendix 2 Decision curve analysis (DCA) for 20 machine learning algorithms in external validation.

## Abbreviations

AI: artificial intelligence
AUC: area under the curve
DCA: decision curve analysis
GEO: Gene Expression Omnibus
KNN: k-nearest neighbors algorithm
LASSO: least absolute shrinkage and selection operator
ML: machine learning
RF: random forest
RFE: recursive feature elimination
ROC: receiver operating characteristic
VTE: venous thromboembolism
