# Supplementary figures and images for: Development of Venous Thromboembolism Risk Prediction Models Based on Whole Blood Gene Expression Profiling Using 20 Machine Learning Algorithms: Comprehensive Analysis Study
Source: JMIR Med Inform. 2026 Jan 16;14:e75565. doi: 10.2196/75565 (PMC12810949; doi:10.2196/75565)

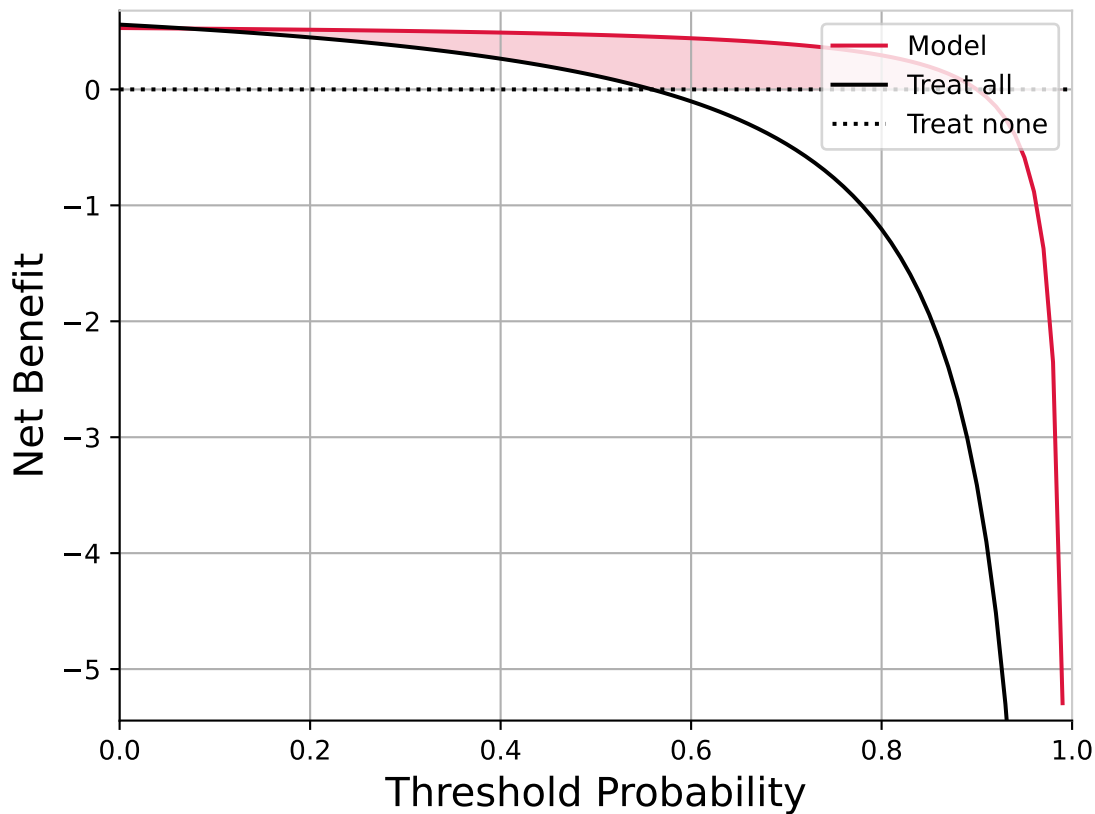

Supplement: Multimedia Appendix 1 [file medinform-v14-e75565-s001.zip › AdaBoost_DCA.pdf]

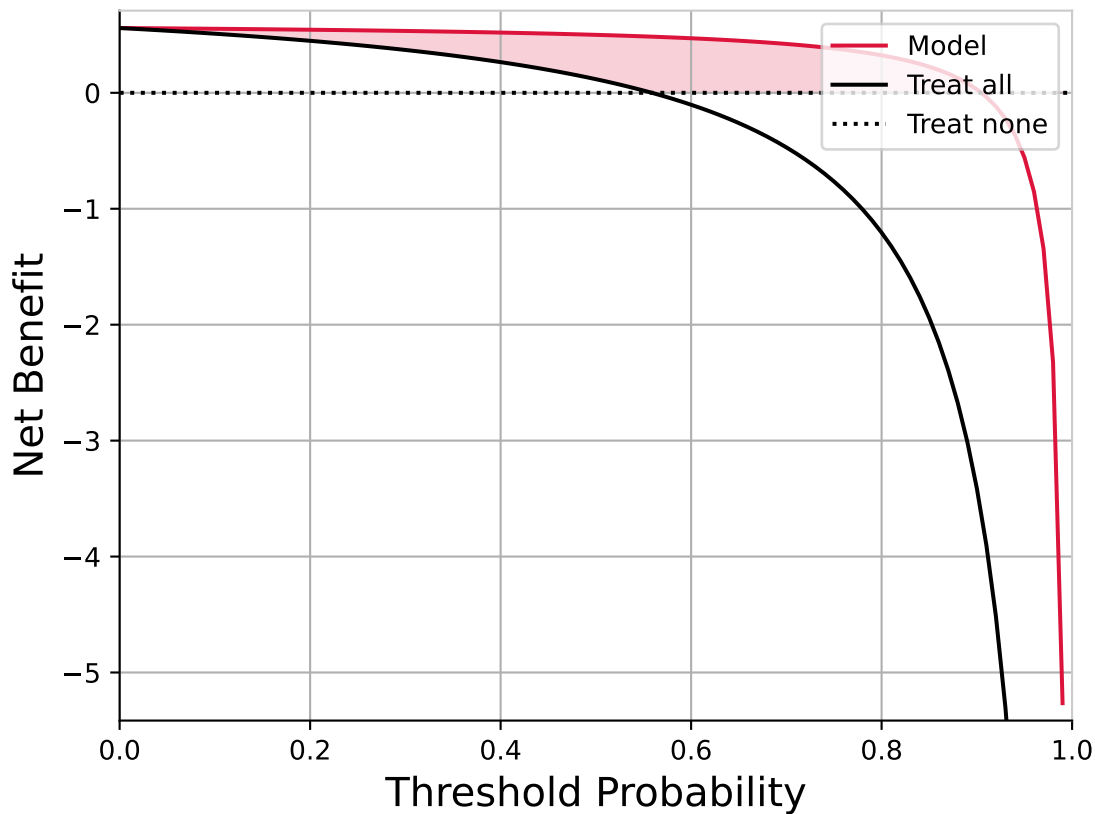

Supplement: Multimedia Appendix 1 [file medinform-v14-e75565-s001.zip › ANN_DCA.pdf]

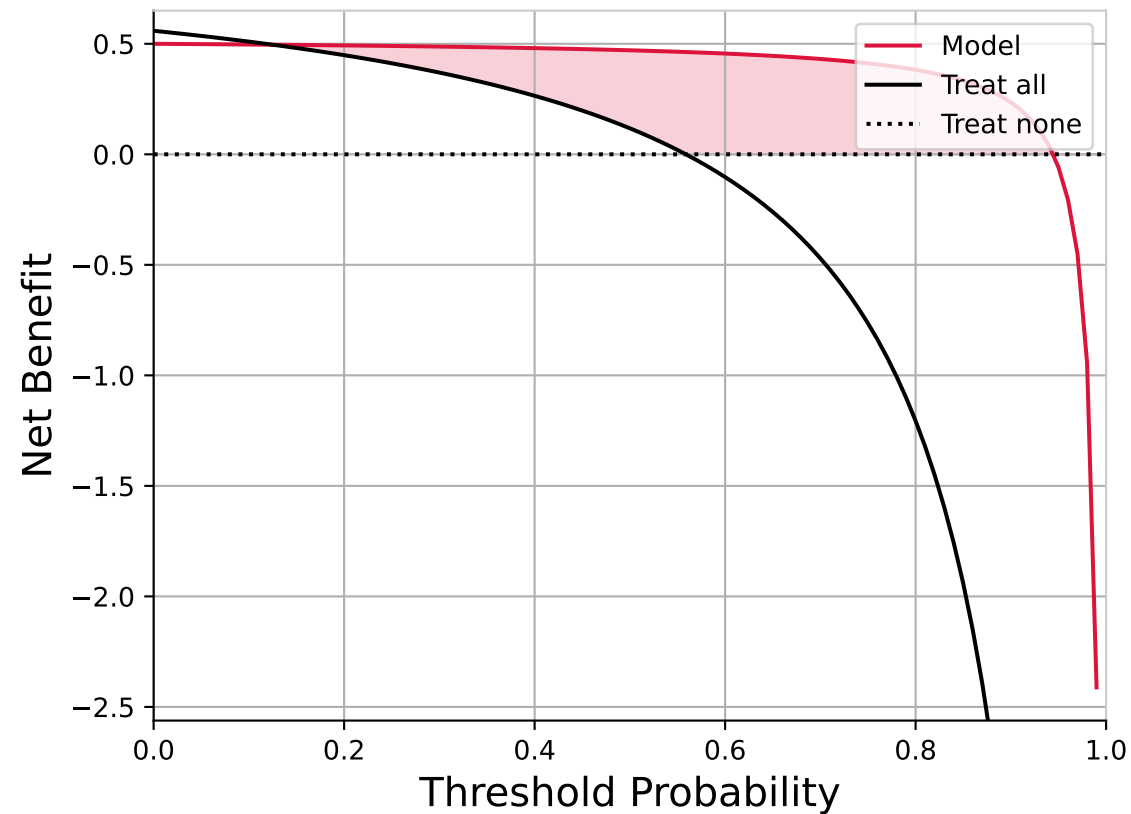

Supplement: Multimedia Appendix 1 [file medinform-v14-e75565-s001.zip › Bagging_DCA.pdf]

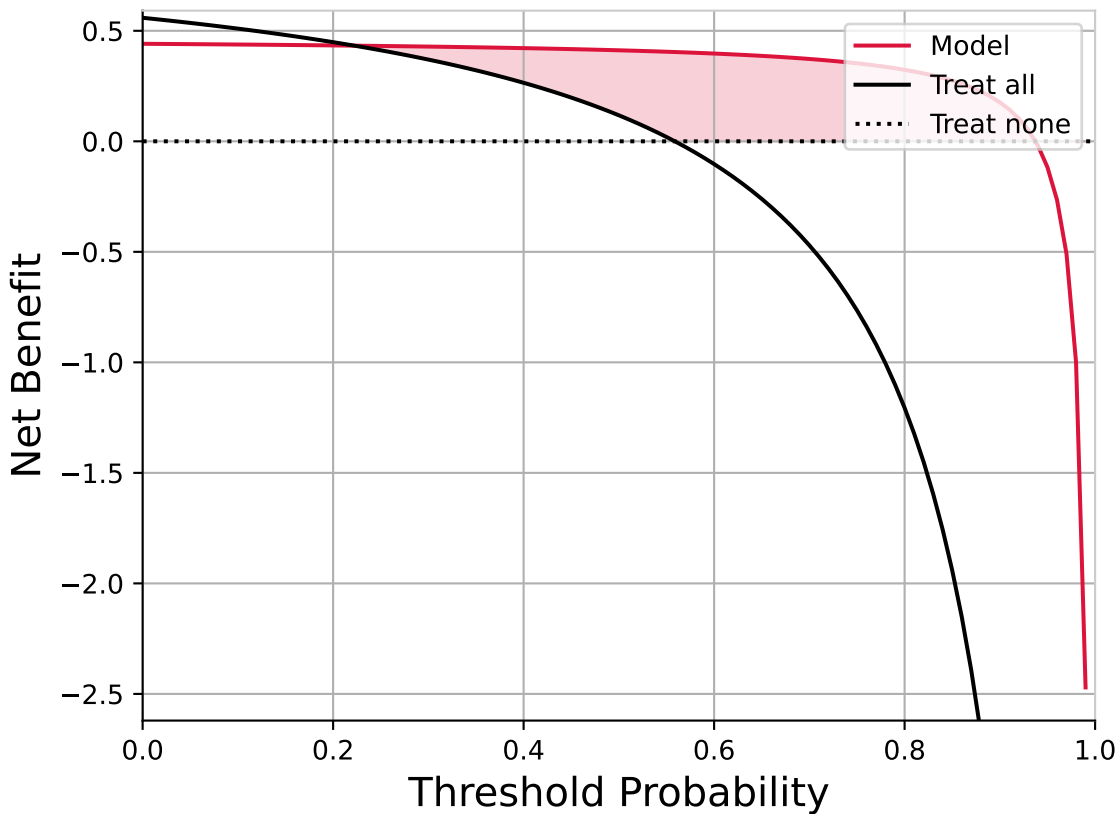

Supplement: Multimedia Appendix 1 [file medinform-v14-e75565-s001.zip › BayesianRidge_DCA.pdf]

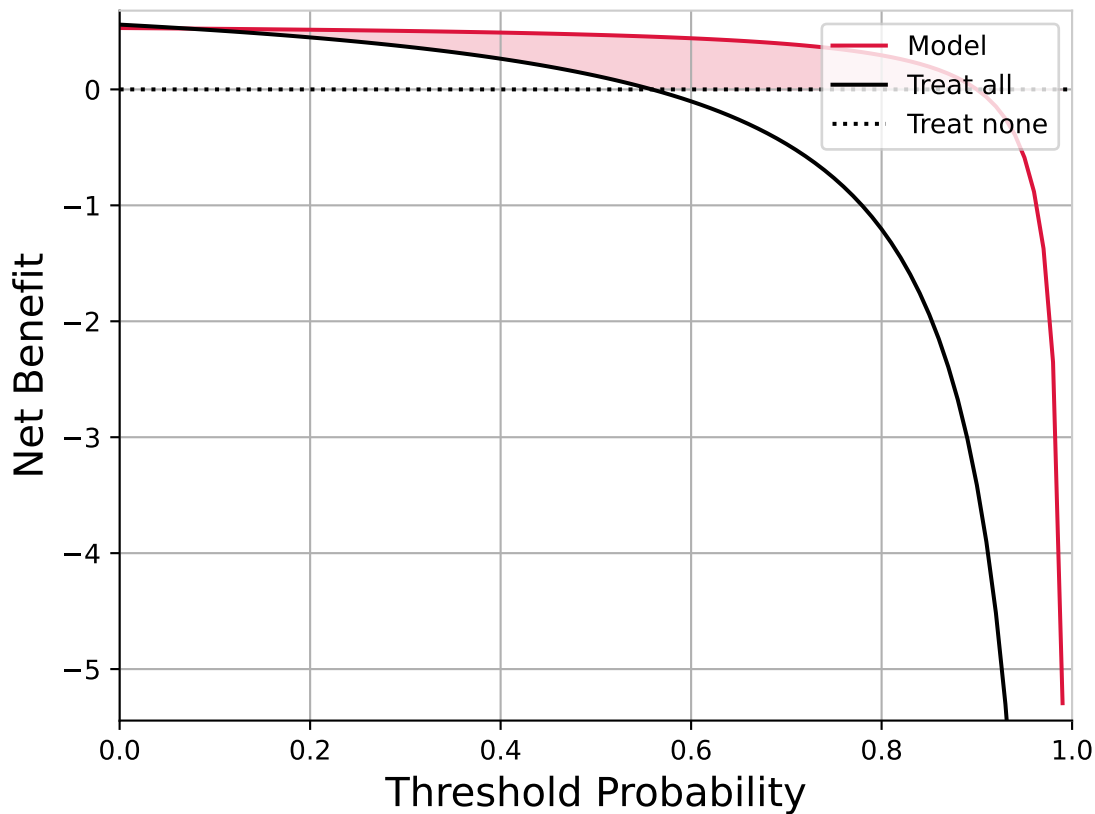

Supplement: Multimedia Appendix 1 [file medinform-v14-e75565-s001.zip › Decision Tree_DCA.pdf]

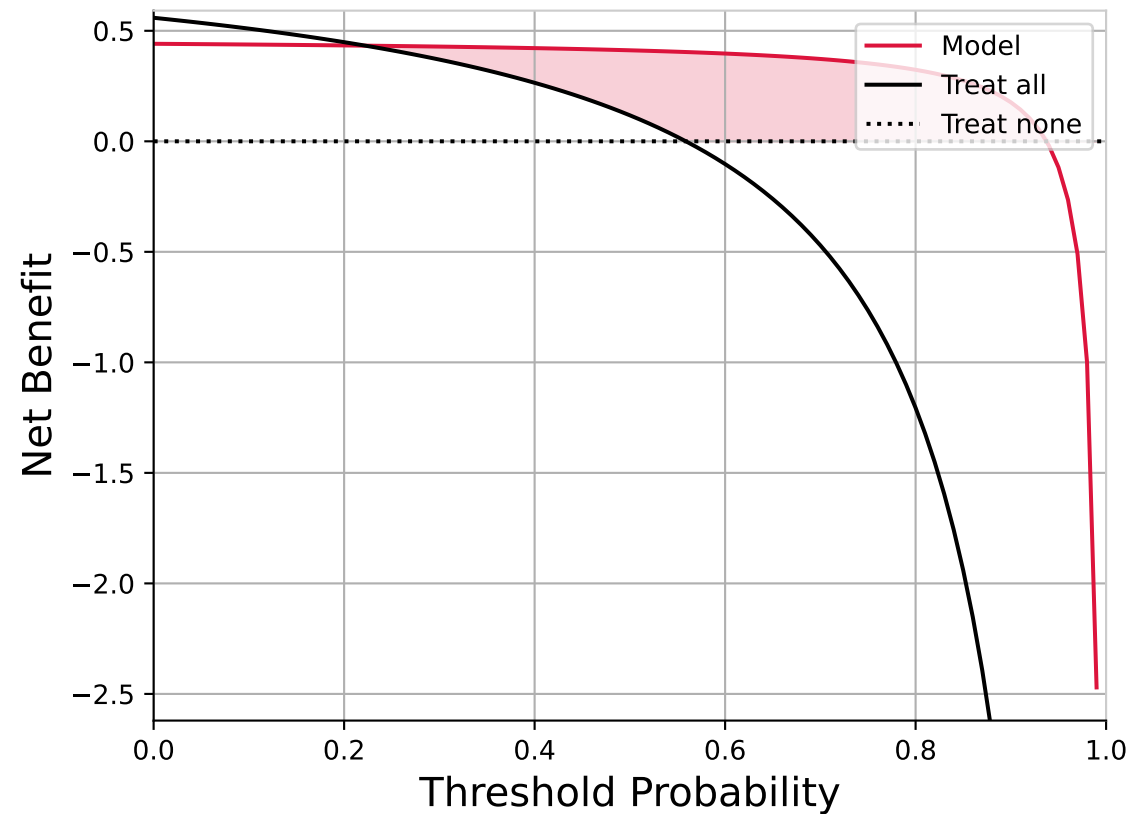

Supplement: Multimedia Appendix 1 [file medinform-v14-e75565-s001.zip › ElasticNet_DCA.pdf]

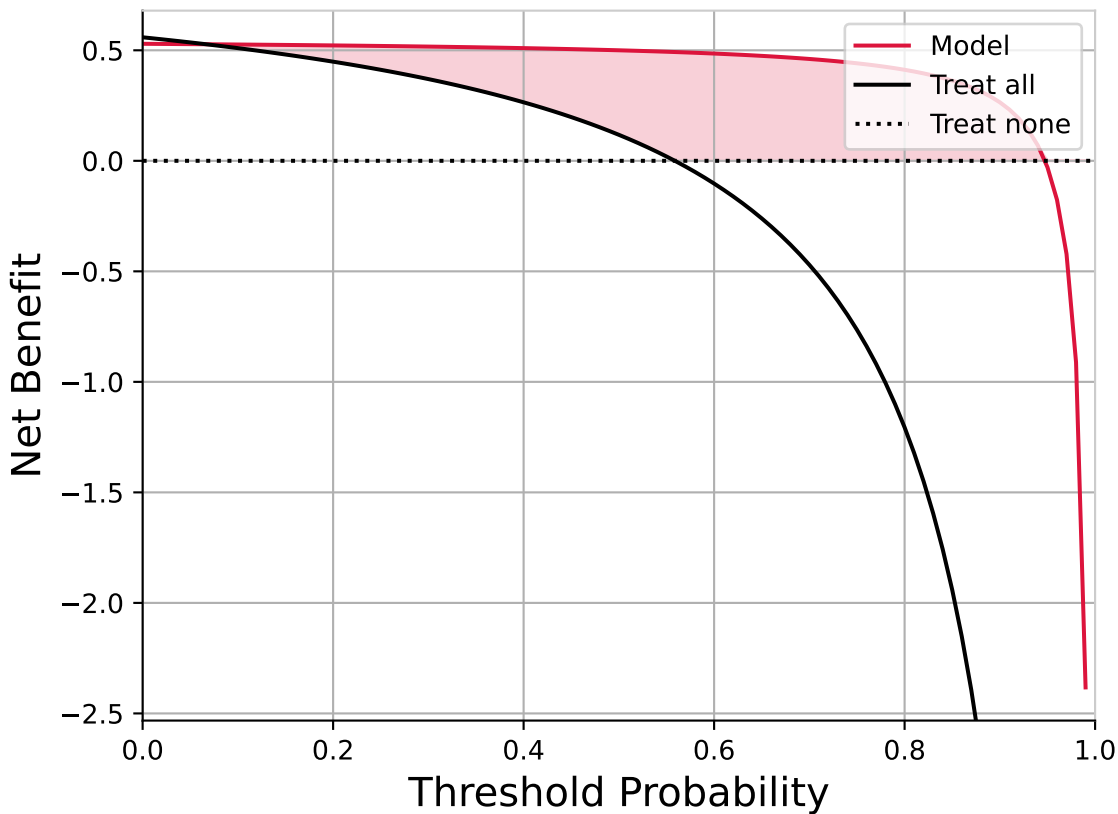

Supplement: Multimedia Appendix 1 [file medinform-v14-e75565-s001.zip › Extra Tree_DCA.pdf]

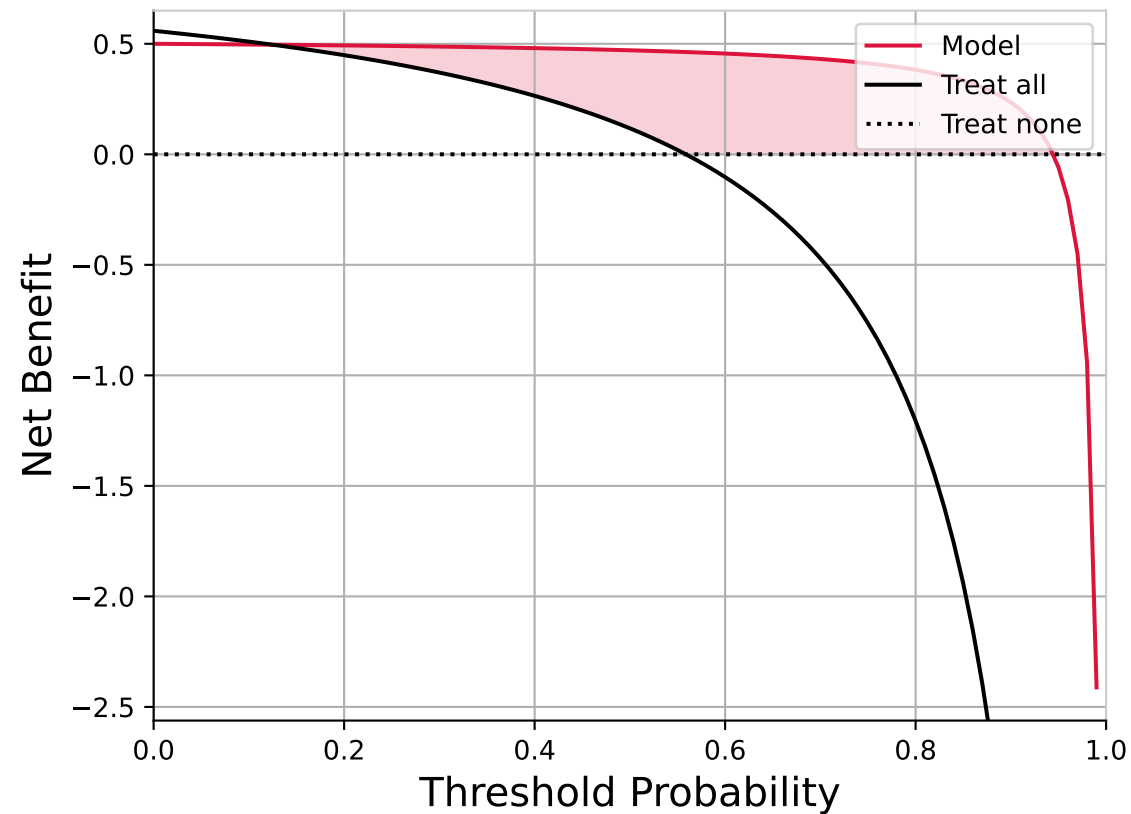

Supplement: Multimedia Appendix 1 [file medinform-v14-e75565-s001.zip › GradientBoosting_DCA.pdf]

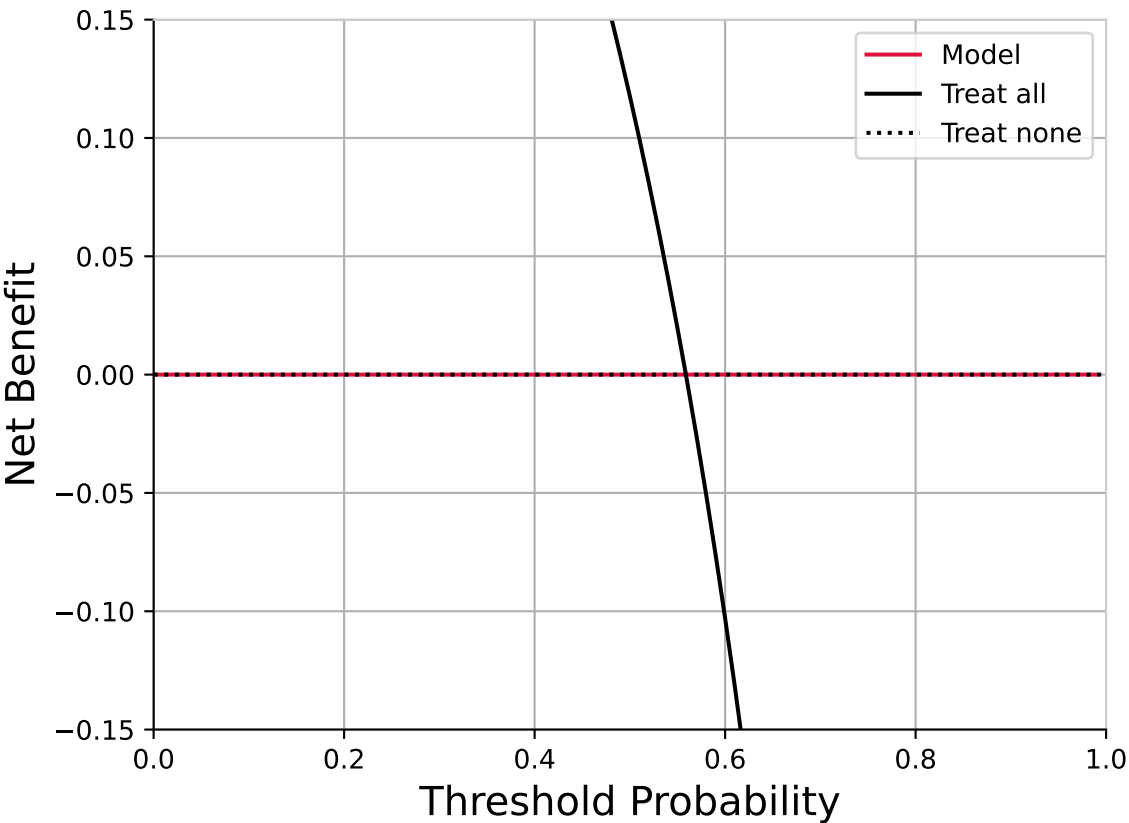

Supplement: Multimedia Appendix 1 [file medinform-v14-e75565-s001.zip › KNN_DCA.pdf]

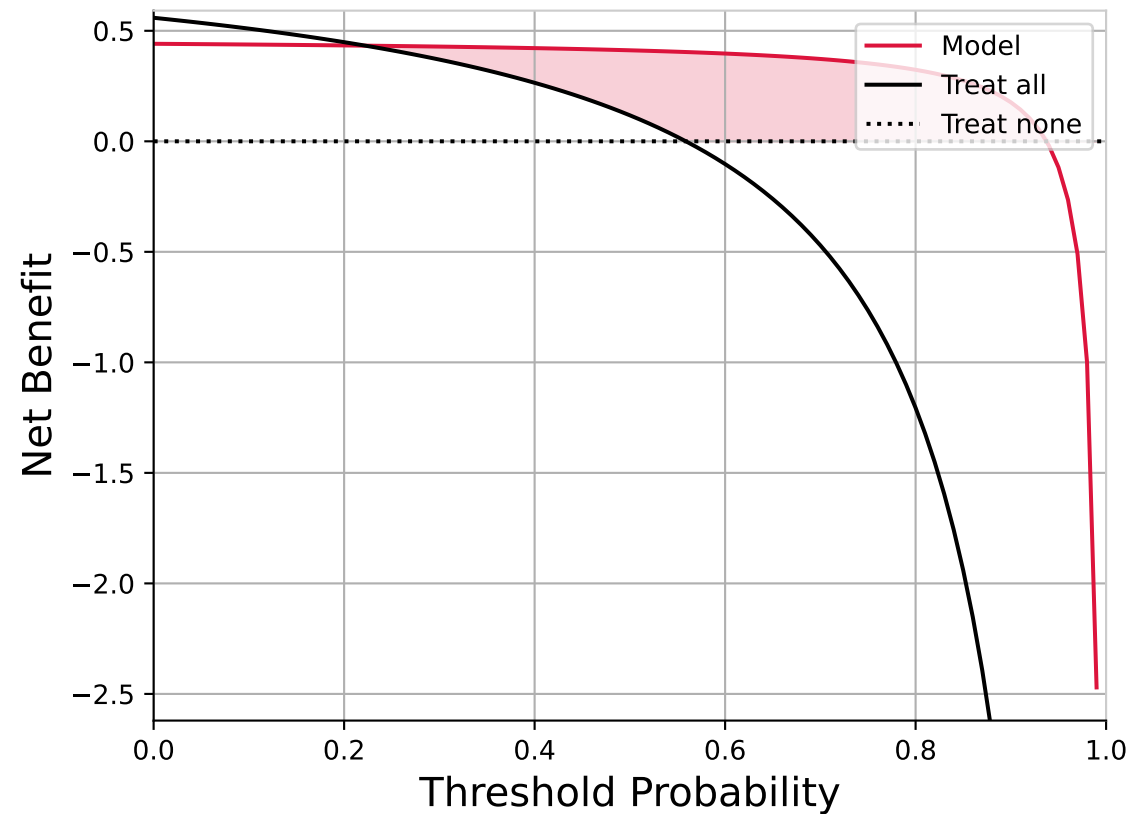

Supplement: Multimedia Appendix 1 [file medinform-v14-e75565-s001.zip › Lasso_DCA.pdf]

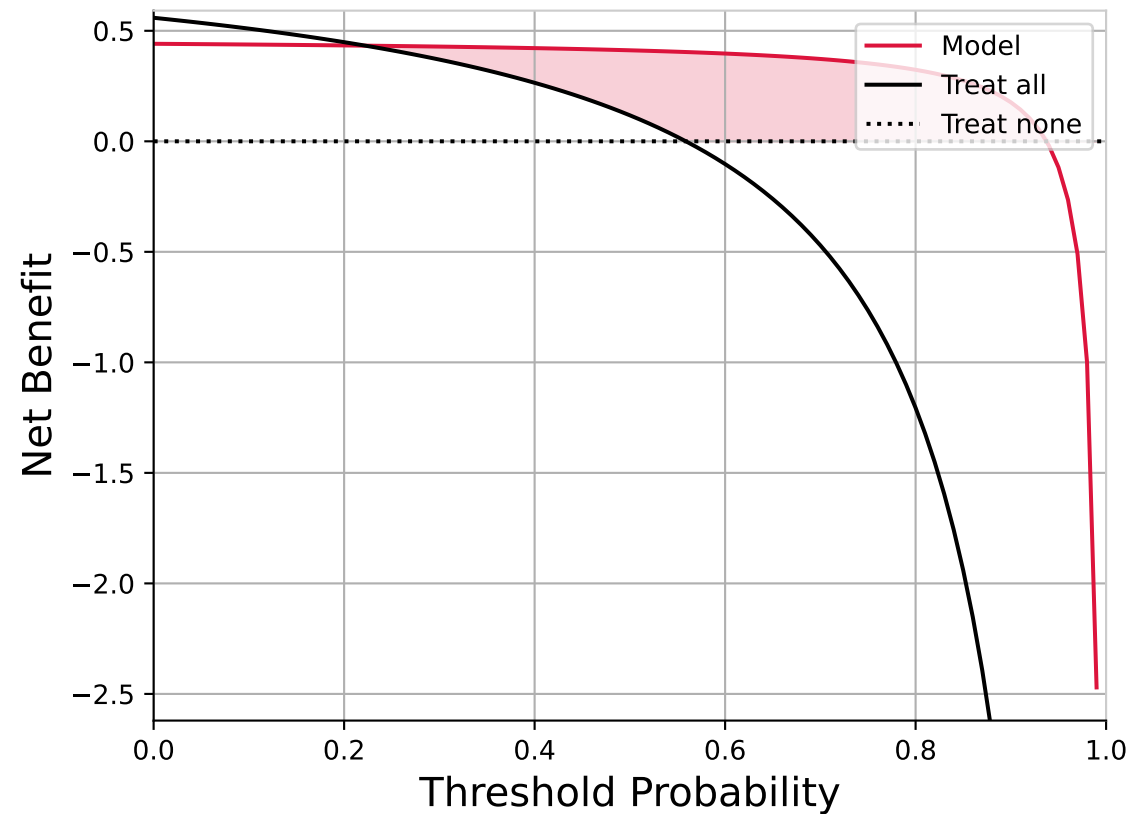

Supplement: Multimedia Appendix 1 [file medinform-v14-e75565-s001.zip › Linear Lasso_DCA.pdf]

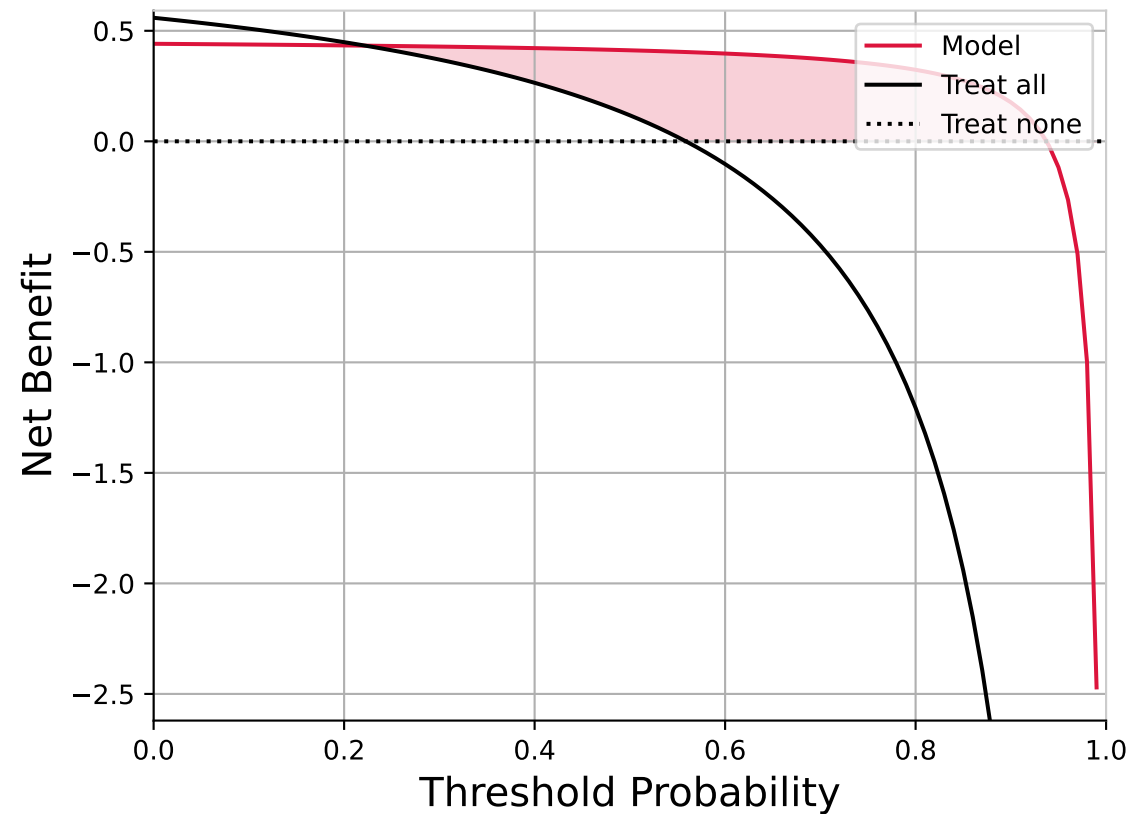

Supplement: Multimedia Appendix 1 [file medinform-v14-e75565-s001.zip › Linear Regression_DCA.pdf]

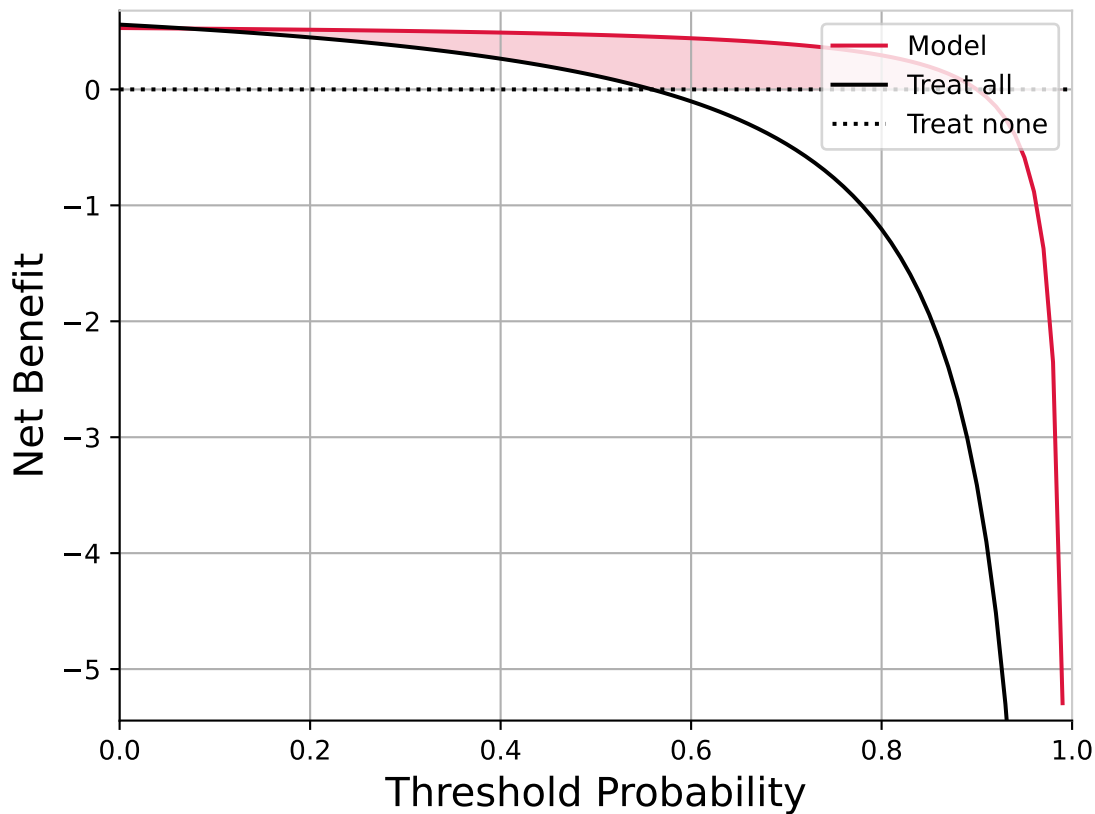

Supplement: Multimedia Appendix 1 [file medinform-v14-e75565-s001.zip › Logistic Regression_DCA.pdf]

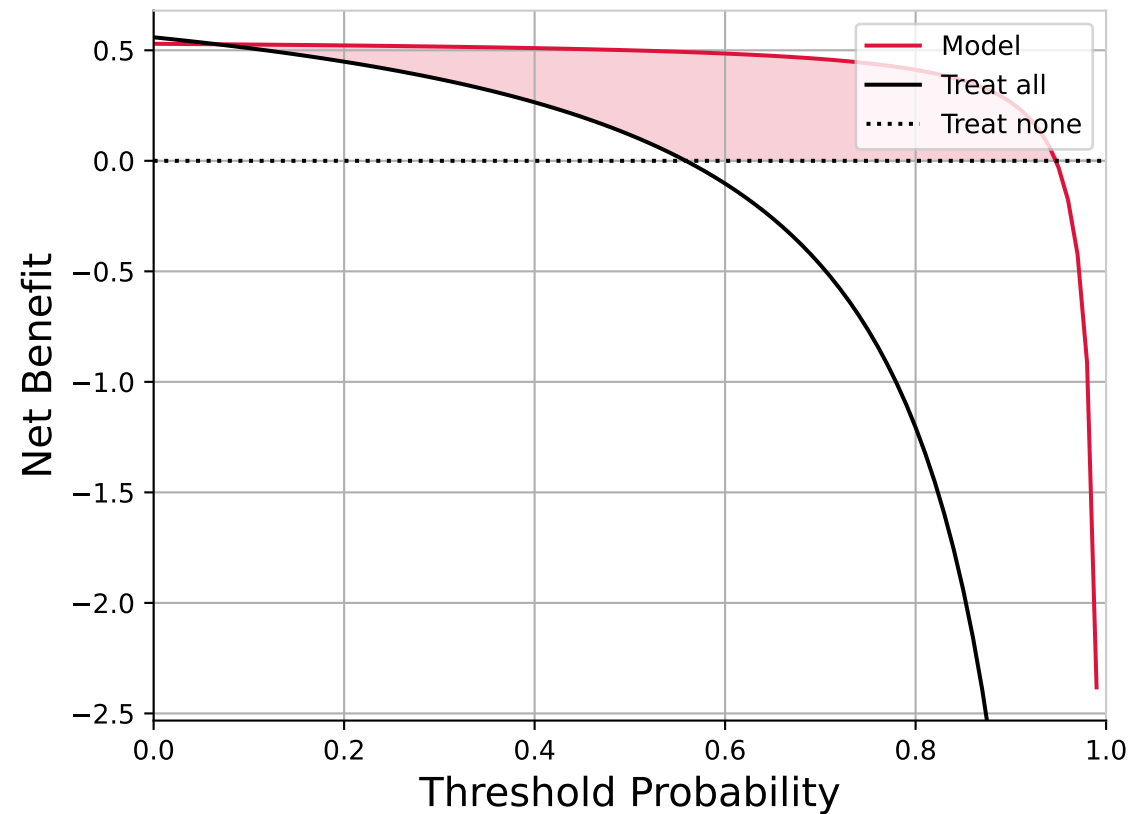

Supplement: Multimedia Appendix 1 [file medinform-v14-e75565-s001.zip › Naive Bayes_DCA.pdf]

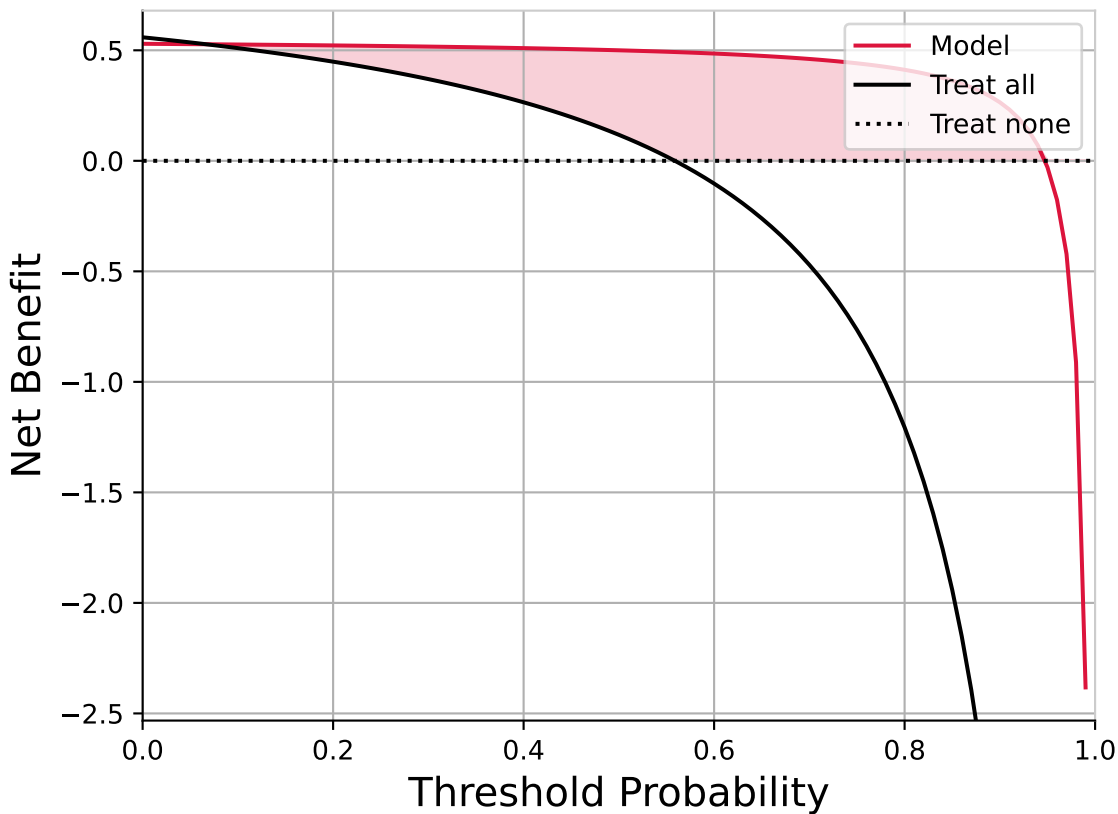

Supplement: Multimedia Appendix 1 [file medinform-v14-e75565-s001.zip › Random Forest_DCA.pdf]

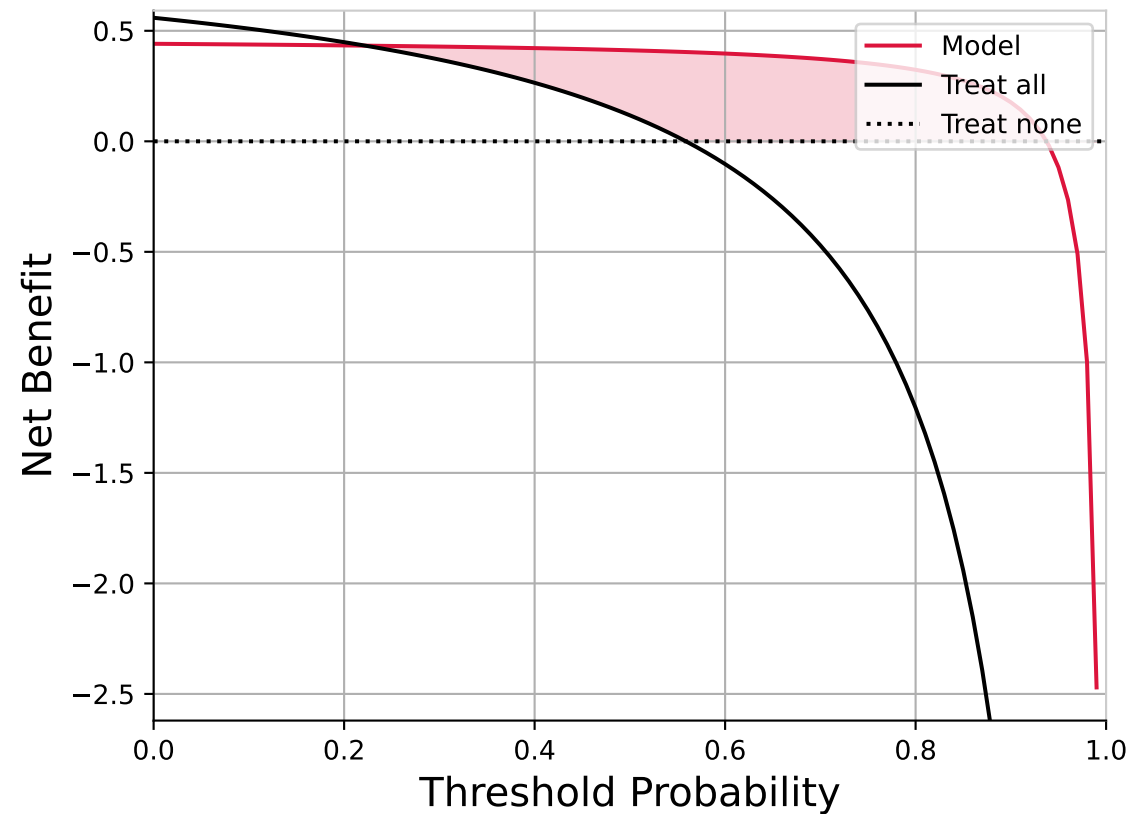

Supplement: Multimedia Appendix 1 [file medinform-v14-e75565-s001.zip › Ridge Regression_DCA.pdf]

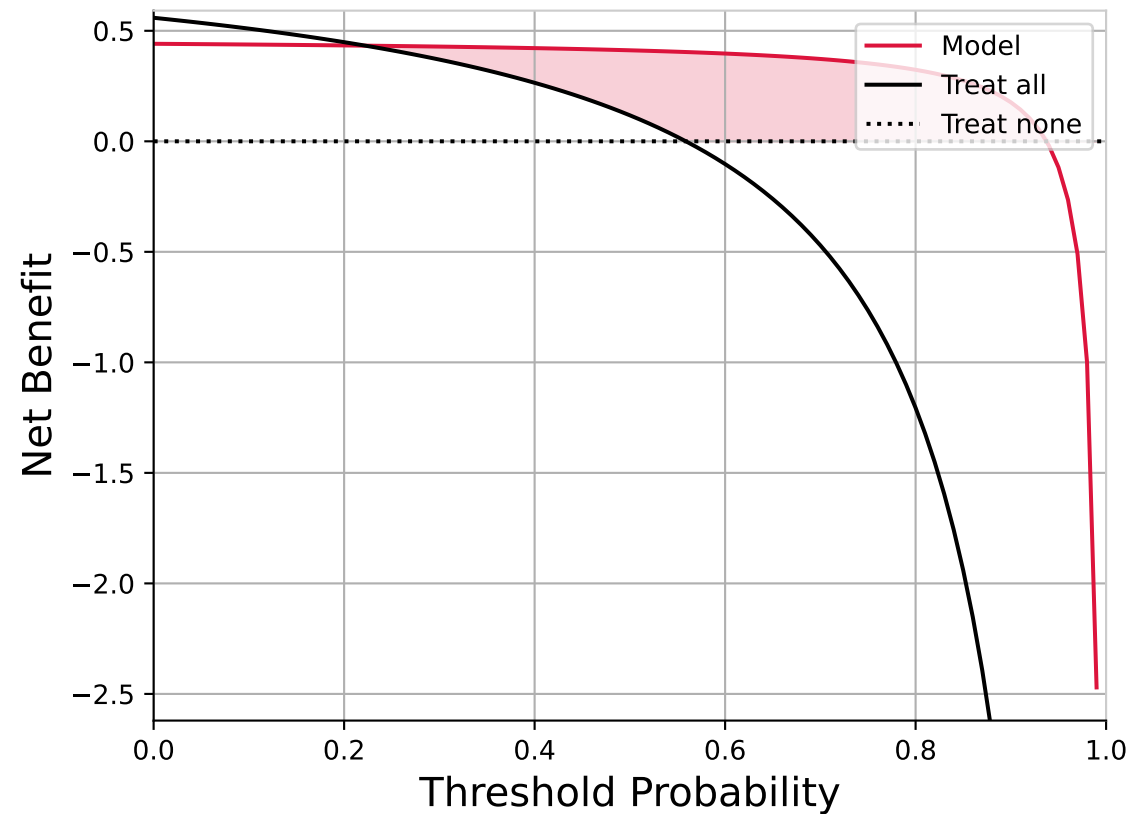

Supplement: Multimedia Appendix 1 [file medinform-v14-e75565-s001.zip › RidgeCV_DCA.pdf]

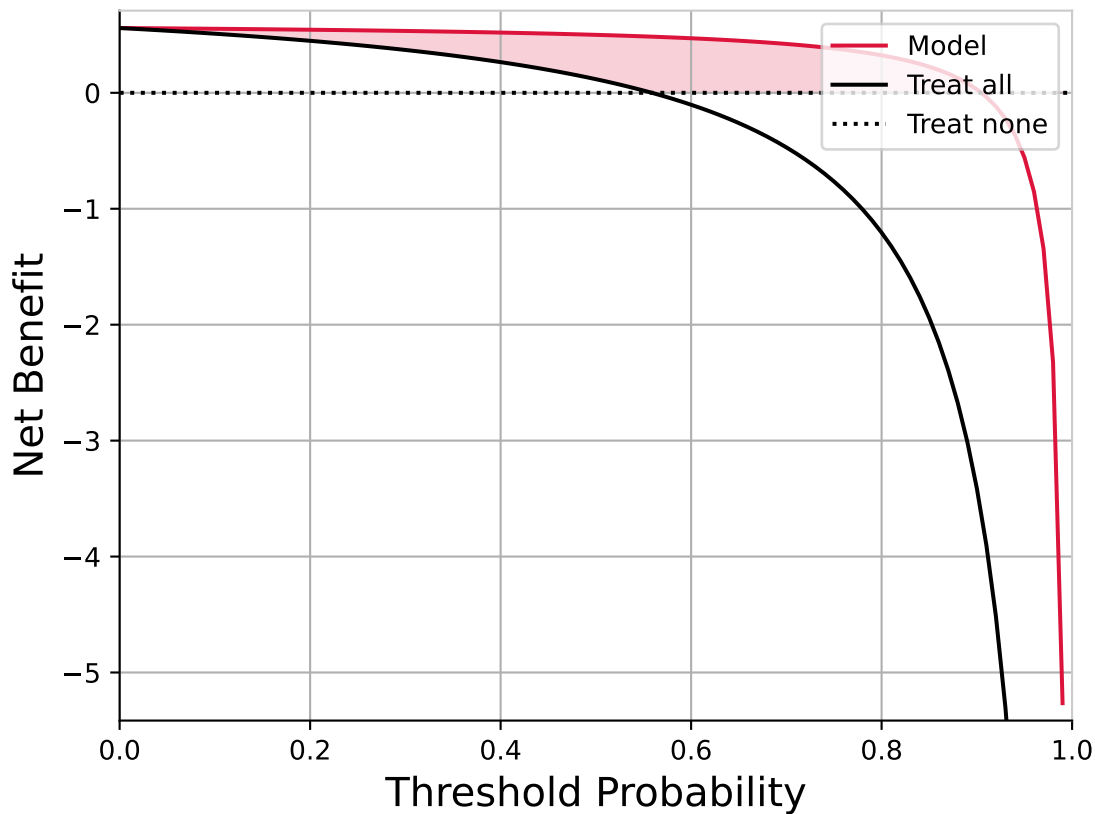

Supplement: Multimedia Appendix 1 [file medinform-v14-e75565-s001.zip › SGD_DCA.pdf]

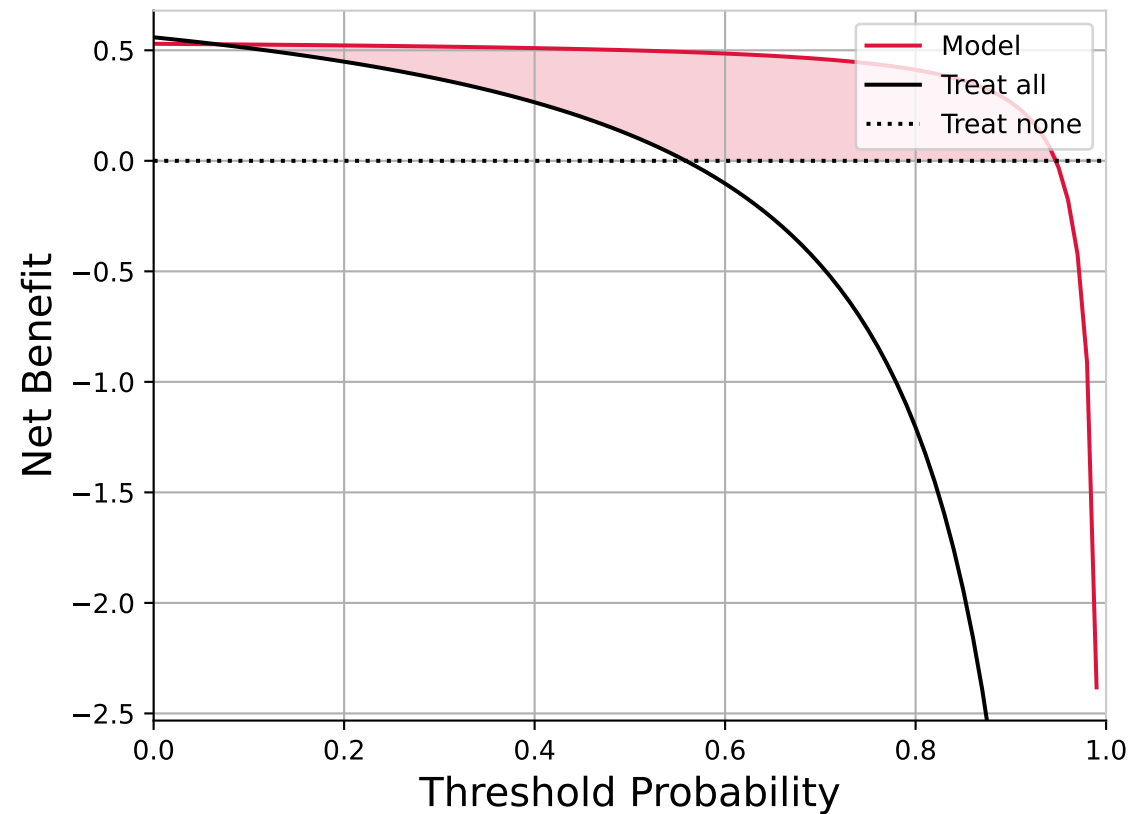

Supplement: Multimedia Appendix 1 [file medinform-v14-e75565-s001.zip › SVM_DCA.pdf]

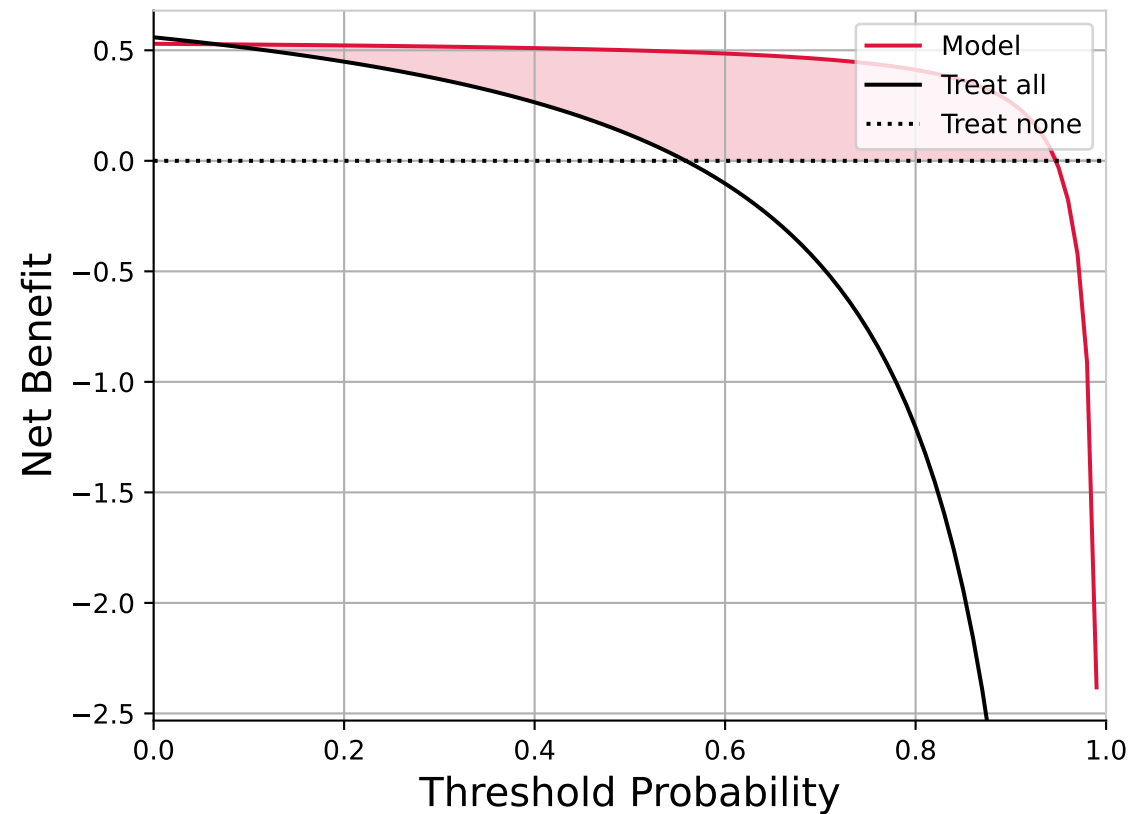

Supplement: Multimedia Appendix 1 [file medinform-v14-e75565-s001.zip › Voting_DCA.pdf]

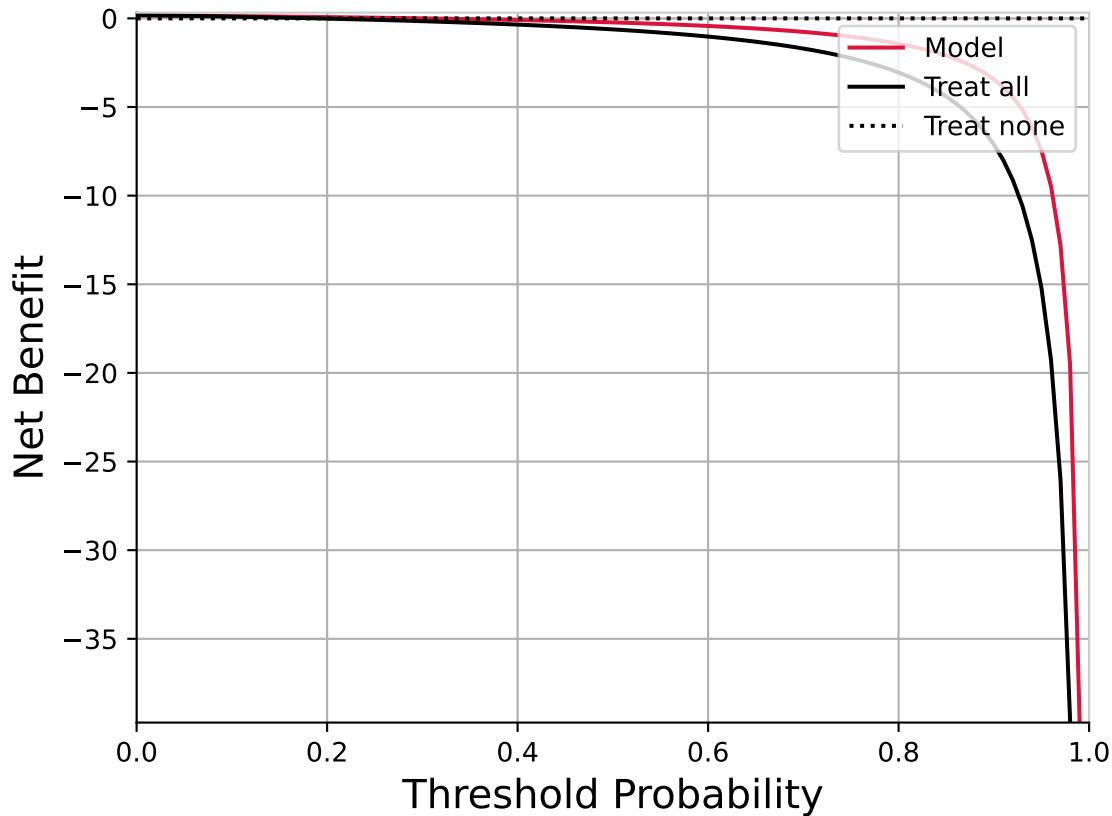

Supplement: Multimedia Appendix 2 [file medinform-v14-e75565-s002.zip › Voting_DCA validation.pdf]

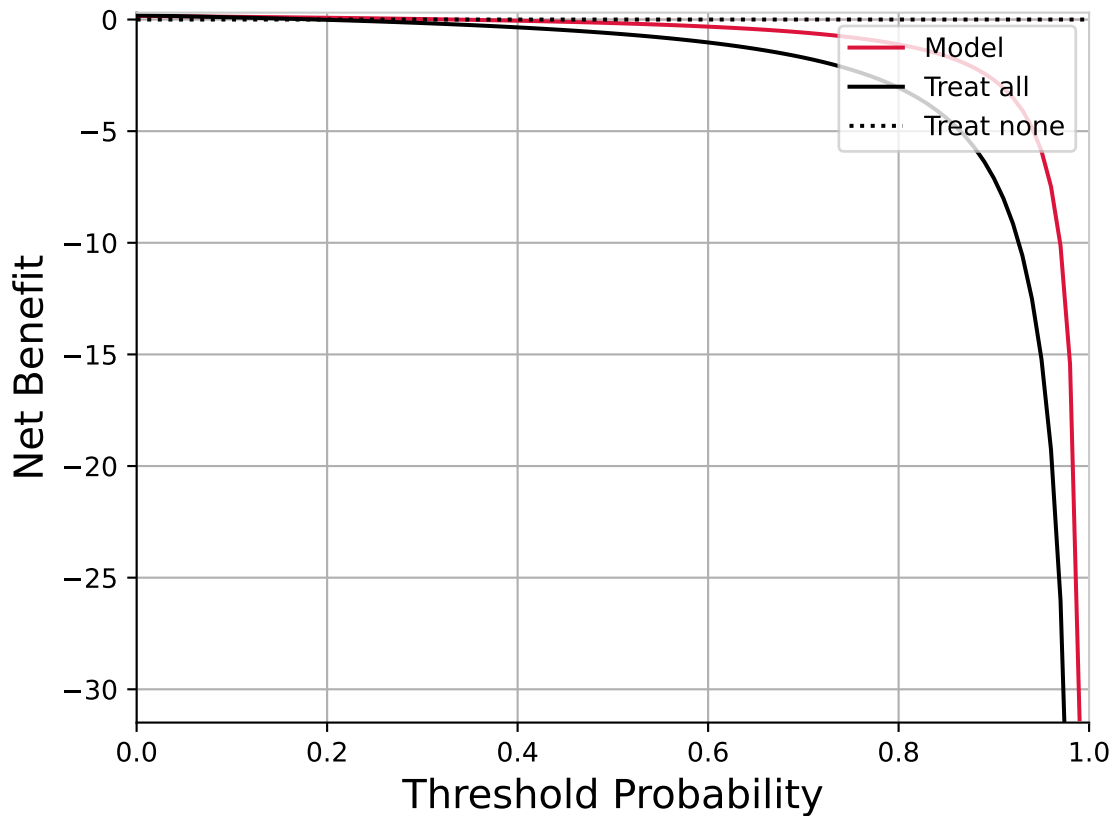

Supplement: Multimedia Appendix 2 [file medinform-v14-e75565-s002.zip › AdaBoost_DCA validation.pdf]

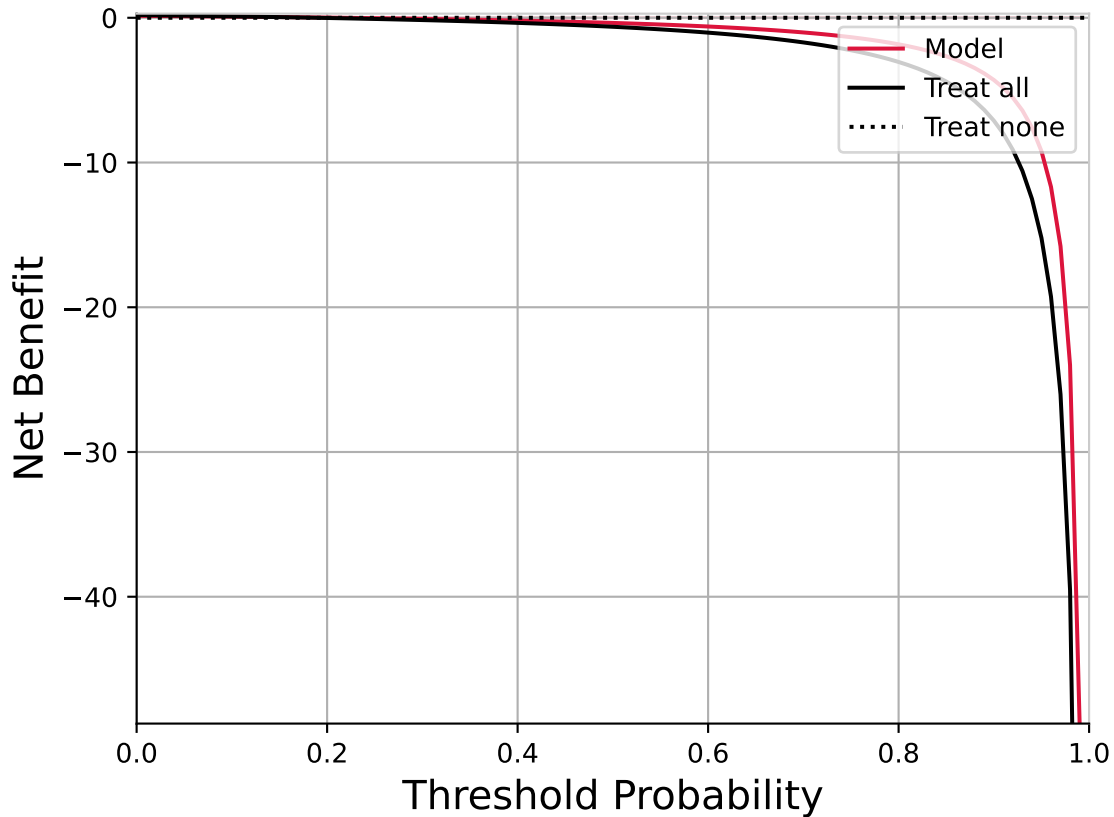

Supplement: Multimedia Appendix 2 [file medinform-v14-e75565-s002.zip › ANN_DCA validation.pdf]

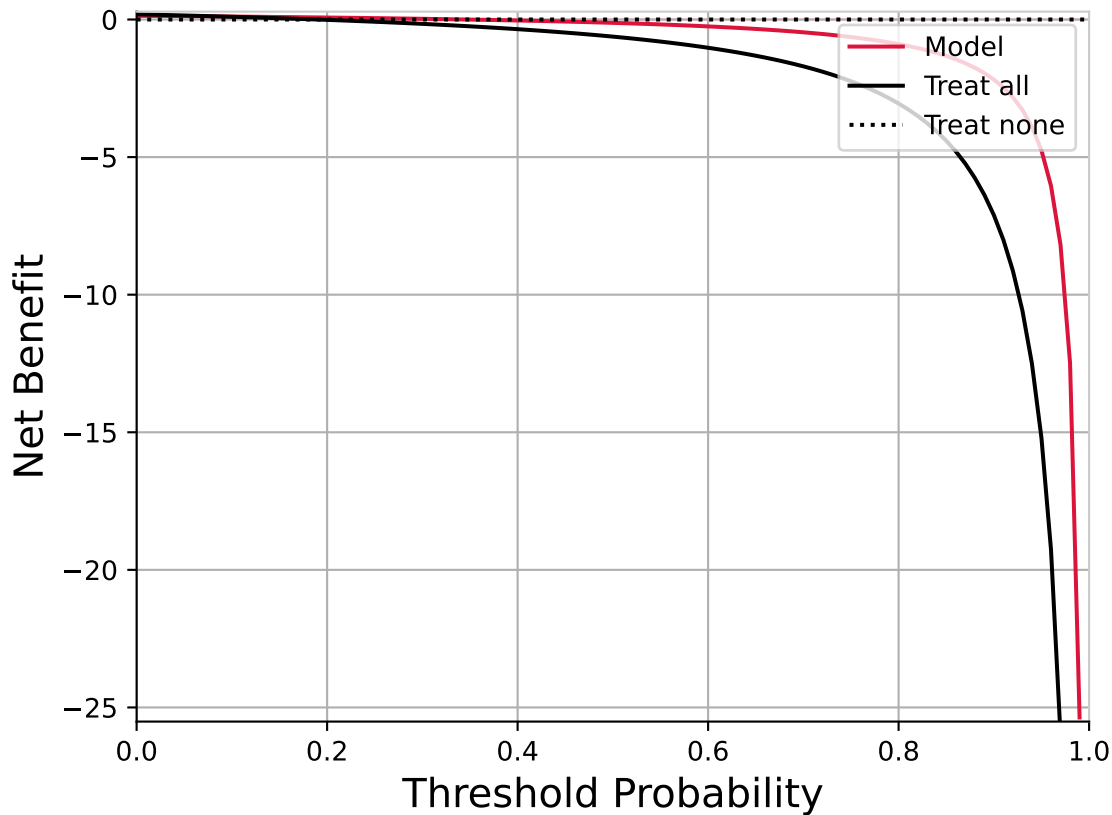

Supplement: Multimedia Appendix 2 [file medinform-v14-e75565-s002.zip › Bagging_DCA validation.pdf]

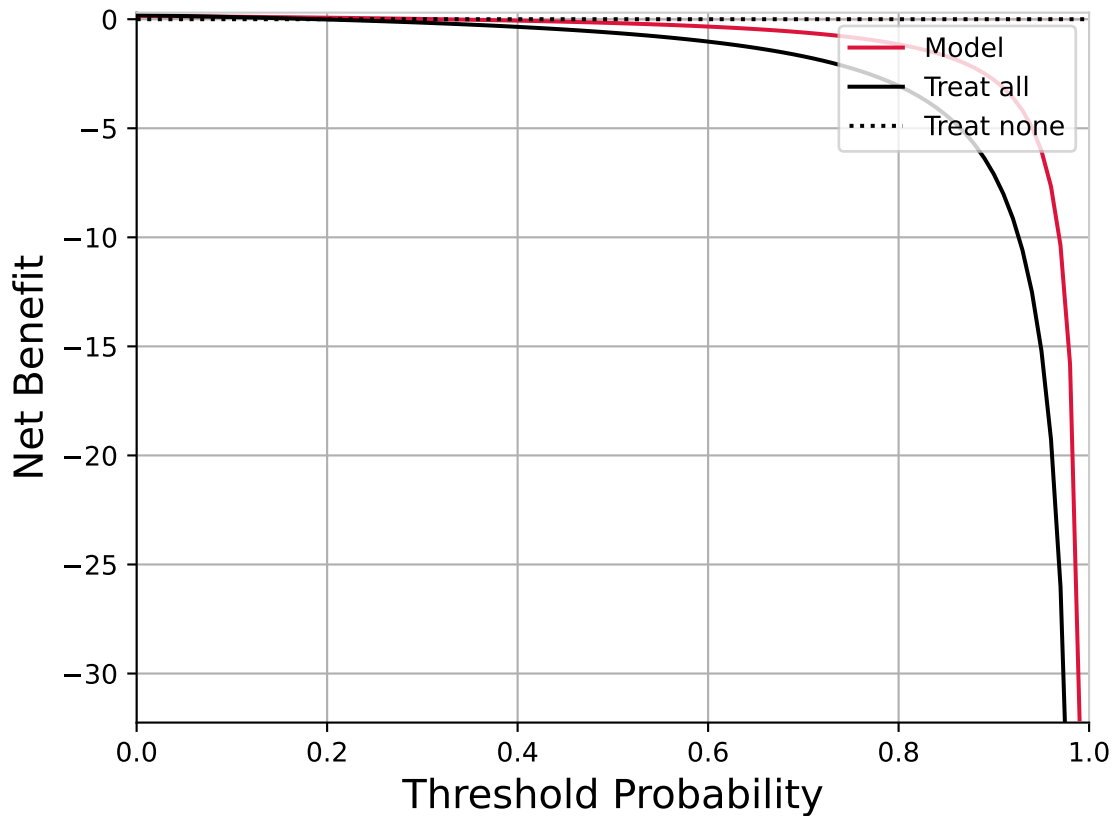

Supplement: Multimedia Appendix 2 [file medinform-v14-e75565-s002.zip › BayesianRidge_DCA validation.pdf]

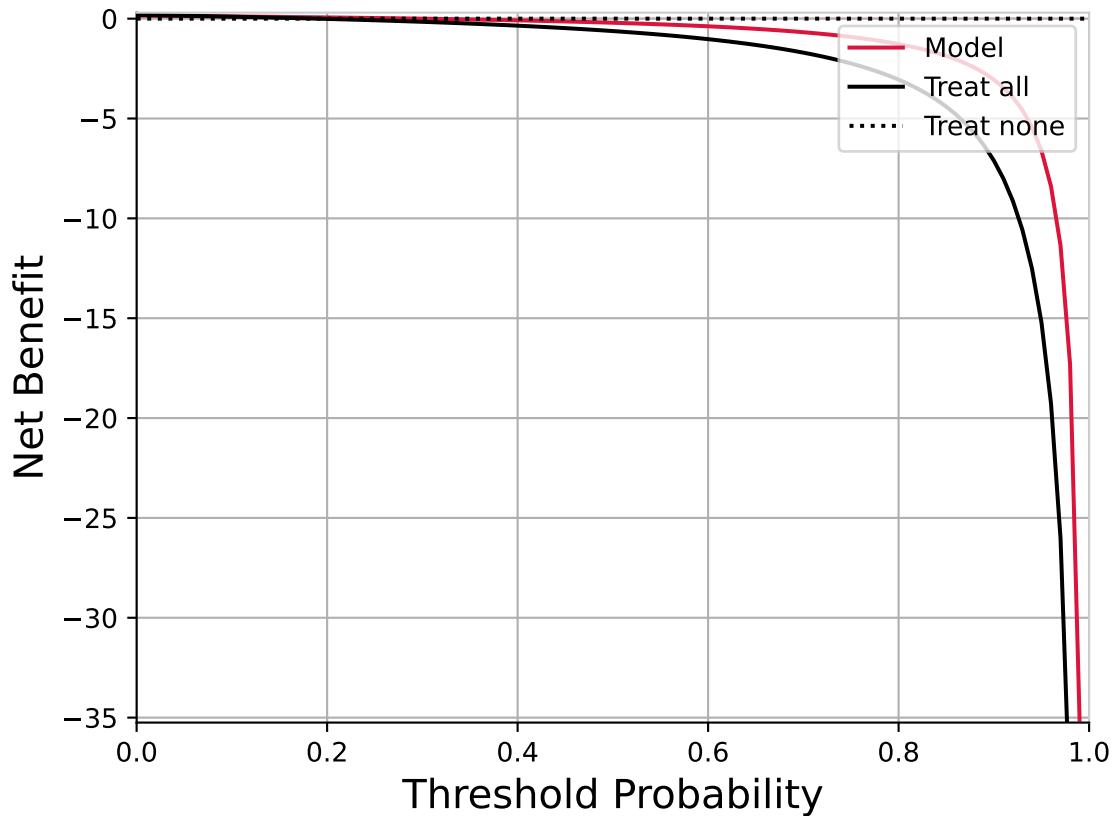

Supplement: Multimedia Appendix 2 [file medinform-v14-e75565-s002.zip › Decision Tree_DCA validation.pdf]

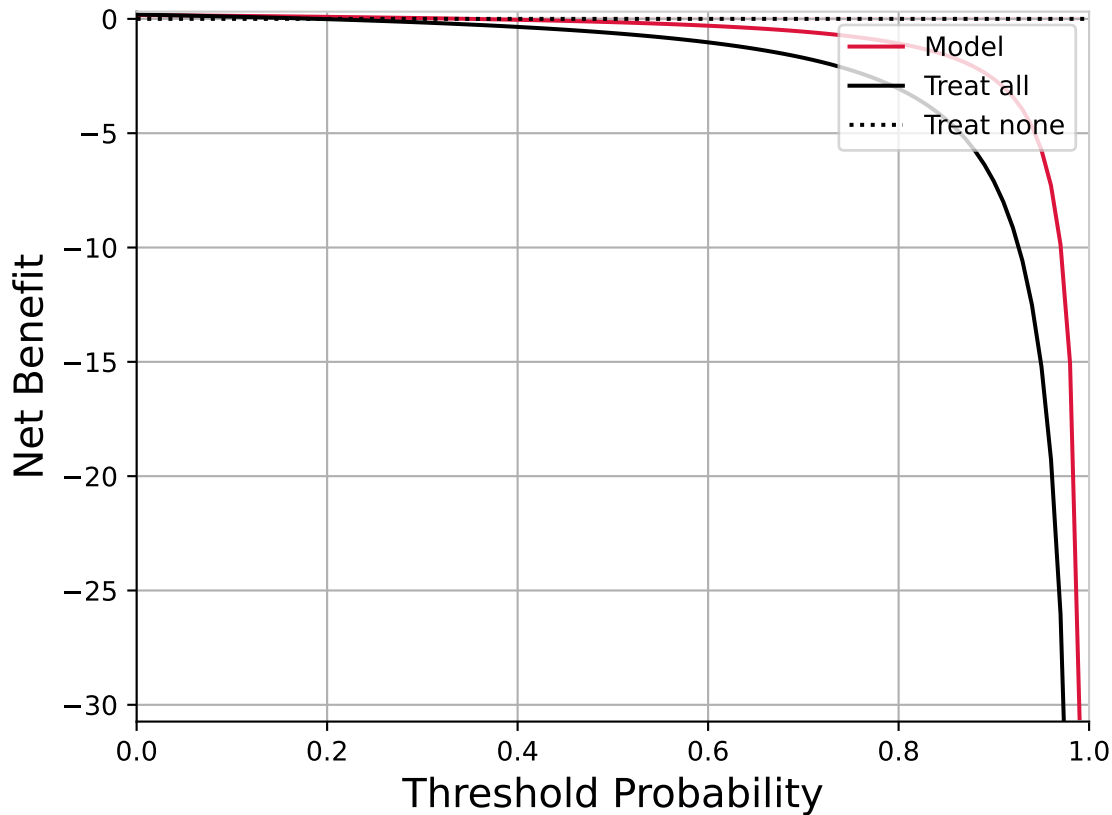

Supplement: Multimedia Appendix 2 [file medinform-v14-e75565-s002.zip › ElasticNet_DCA validation.pdf]

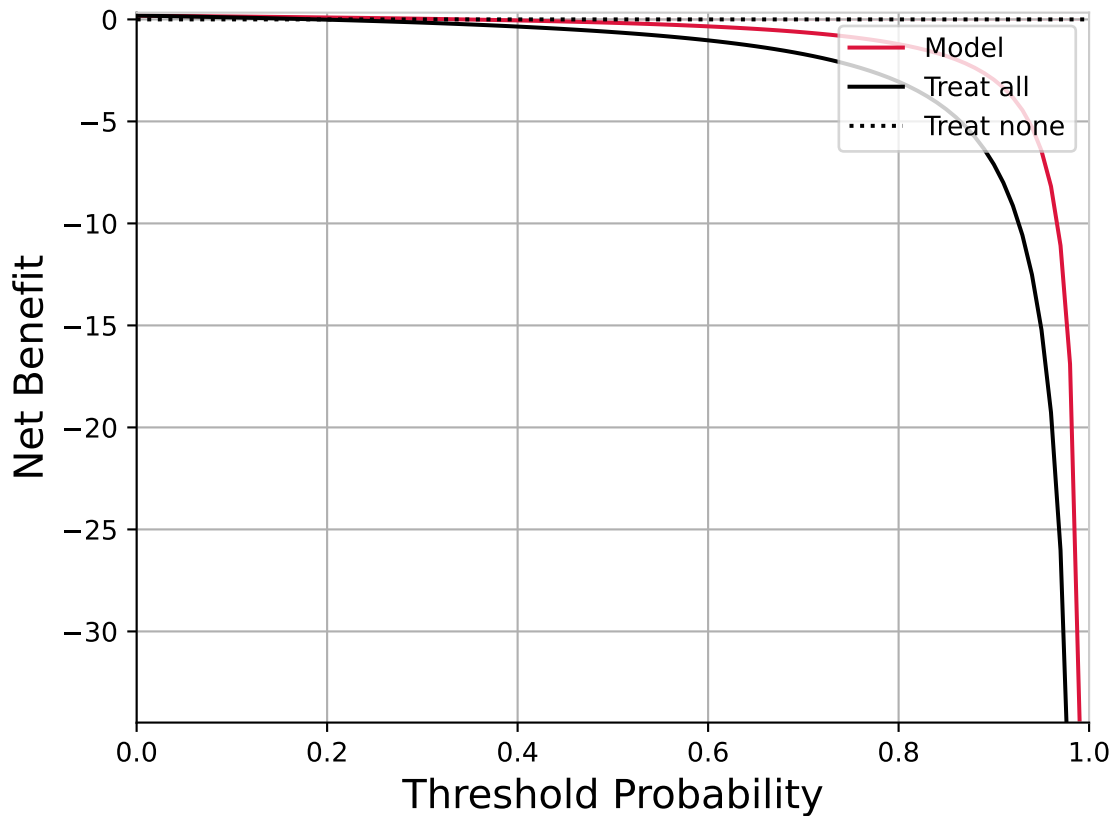

Supplement: Multimedia Appendix 2 [file medinform-v14-e75565-s002.zip › Extra Tree_DCA validation.pdf]

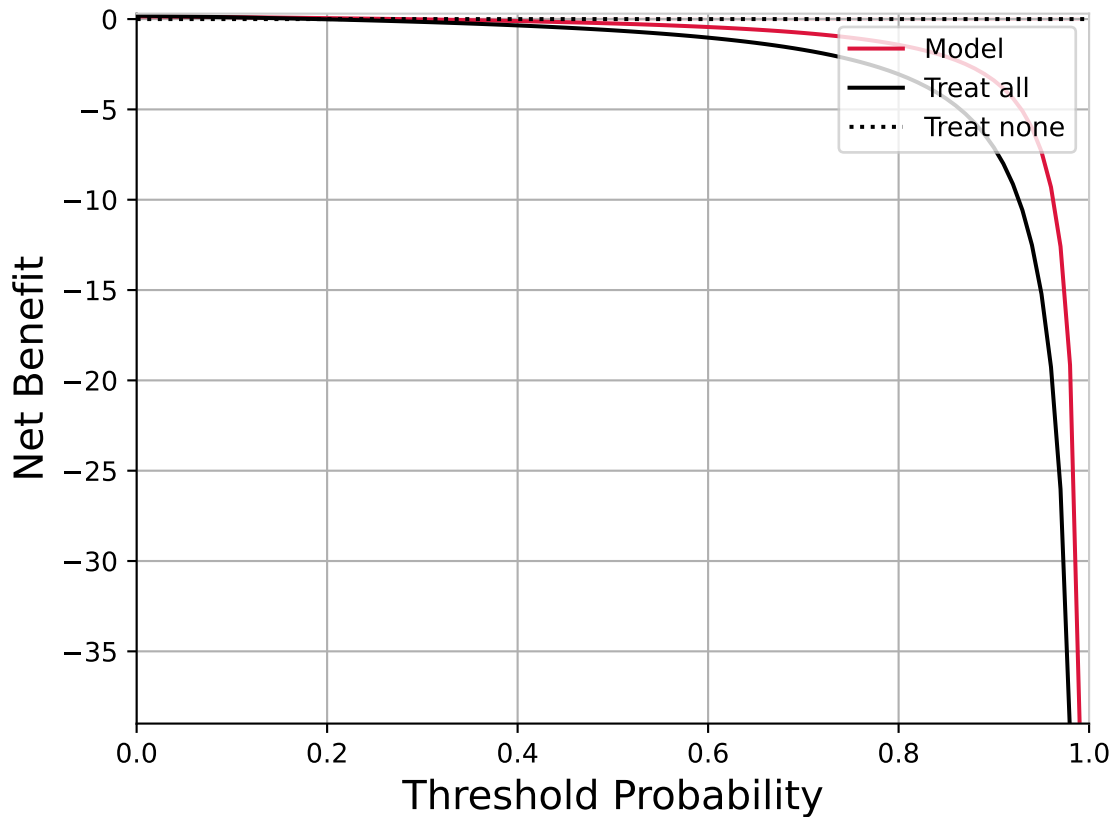

Supplement: Multimedia Appendix 2 [file medinform-v14-e75565-s002.zip › GradientBoosting_DCA validation.pdf]

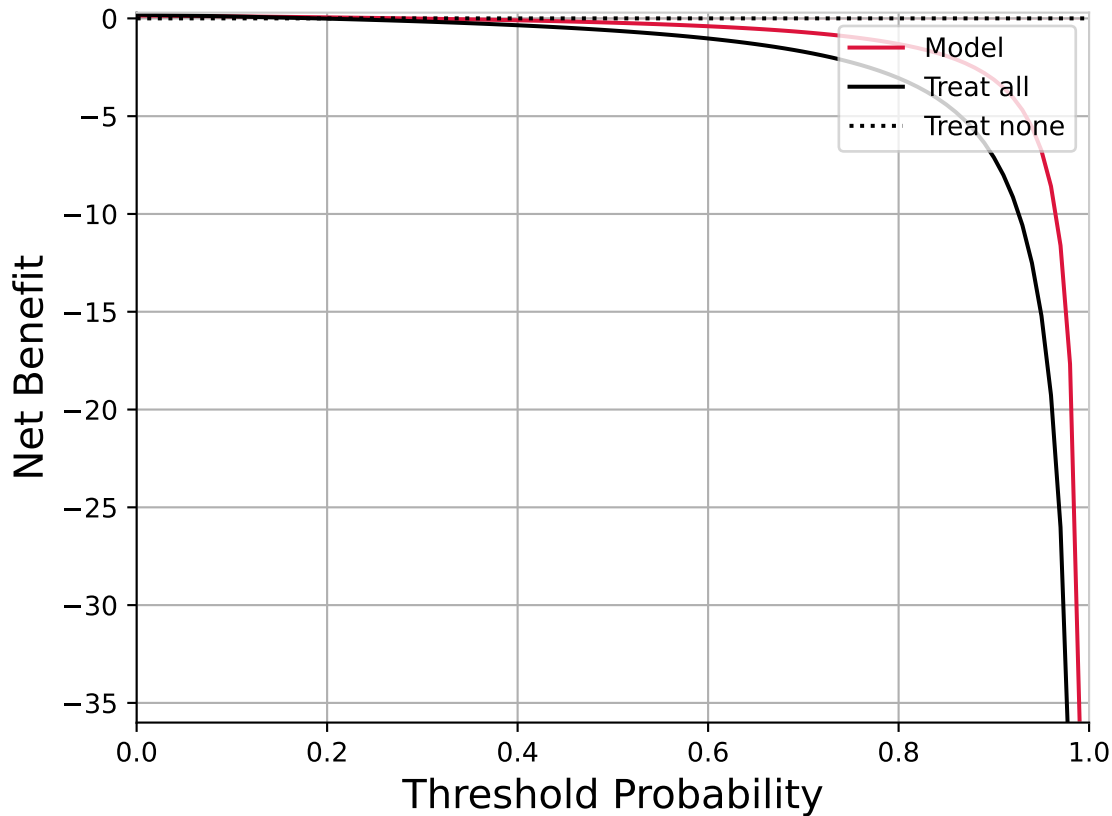

Supplement: Multimedia Appendix 2 [file medinform-v14-e75565-s002.zip › KNN_DCA validation.pdf]

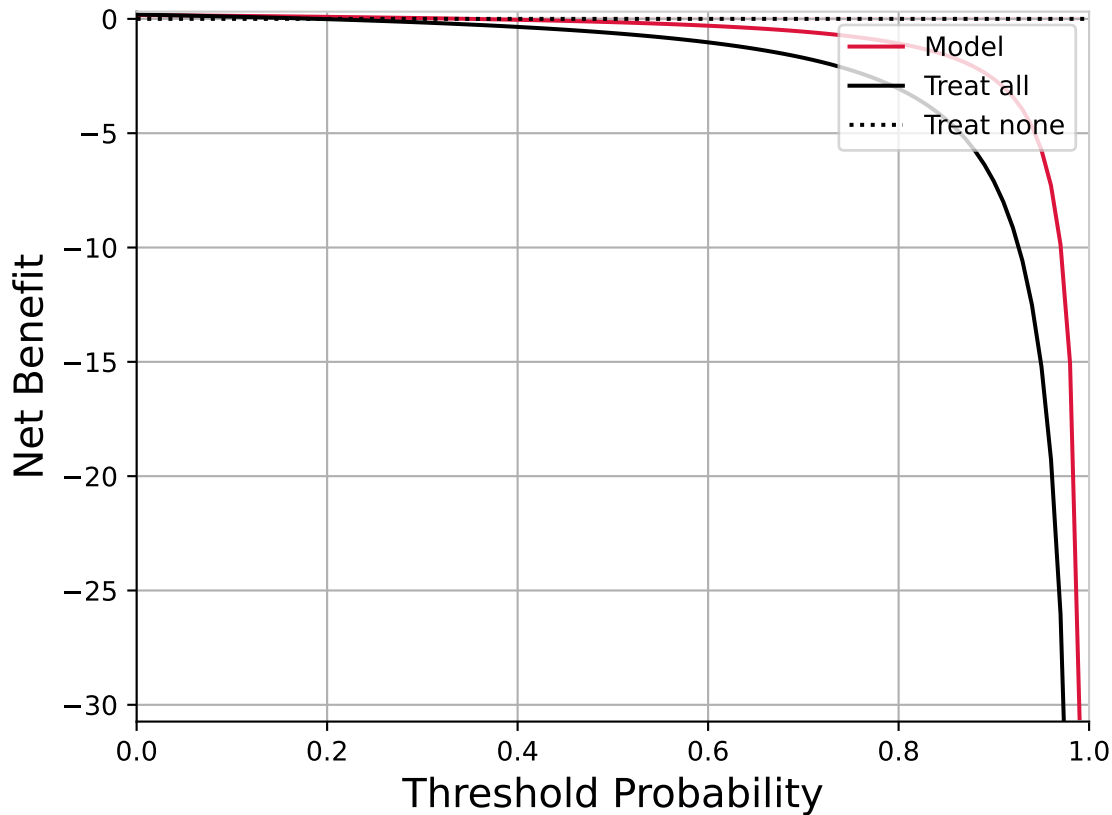

Supplement: Multimedia Appendix 2 [file medinform-v14-e75565-s002.zip › Linear Lasso_DCA validation.pdf]

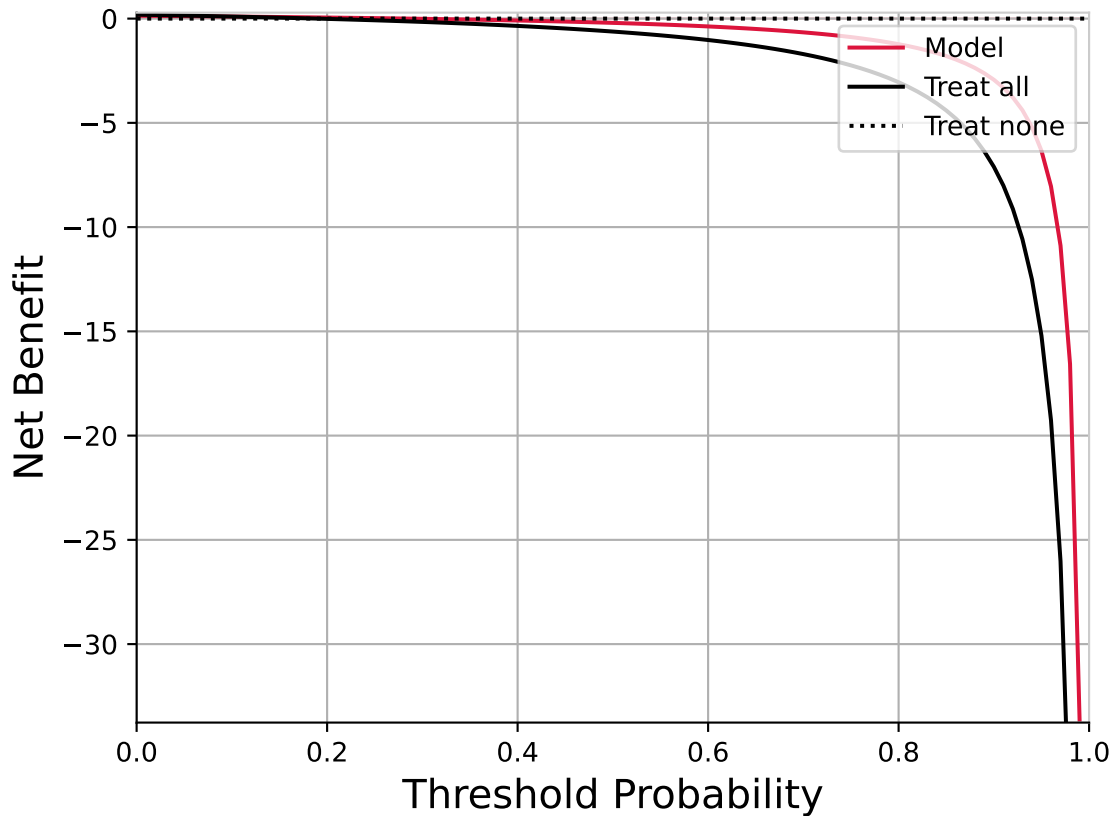

Supplement: Multimedia Appendix 2 [file medinform-v14-e75565-s002.zip › Linear Regression_DCA validation.pdf]

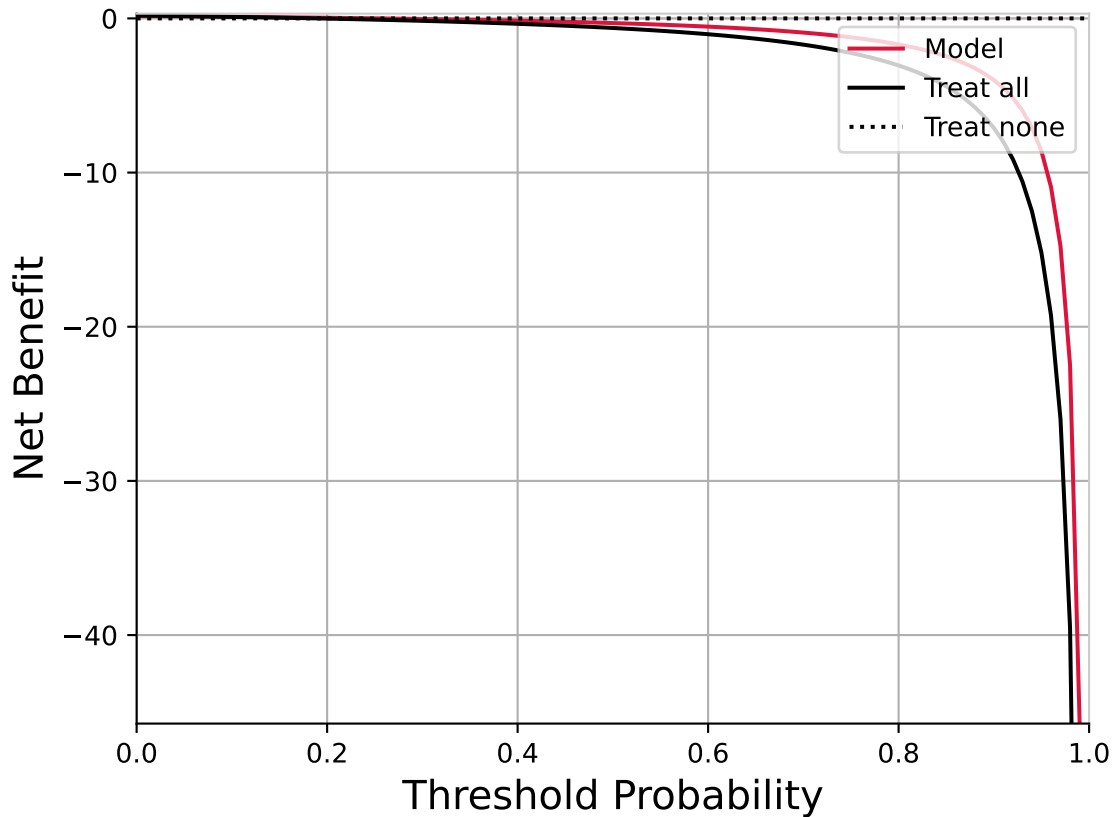

Supplement: Multimedia Appendix 2 [file medinform-v14-e75565-s002.zip › Logistic Regression_DCA validation.pdf]

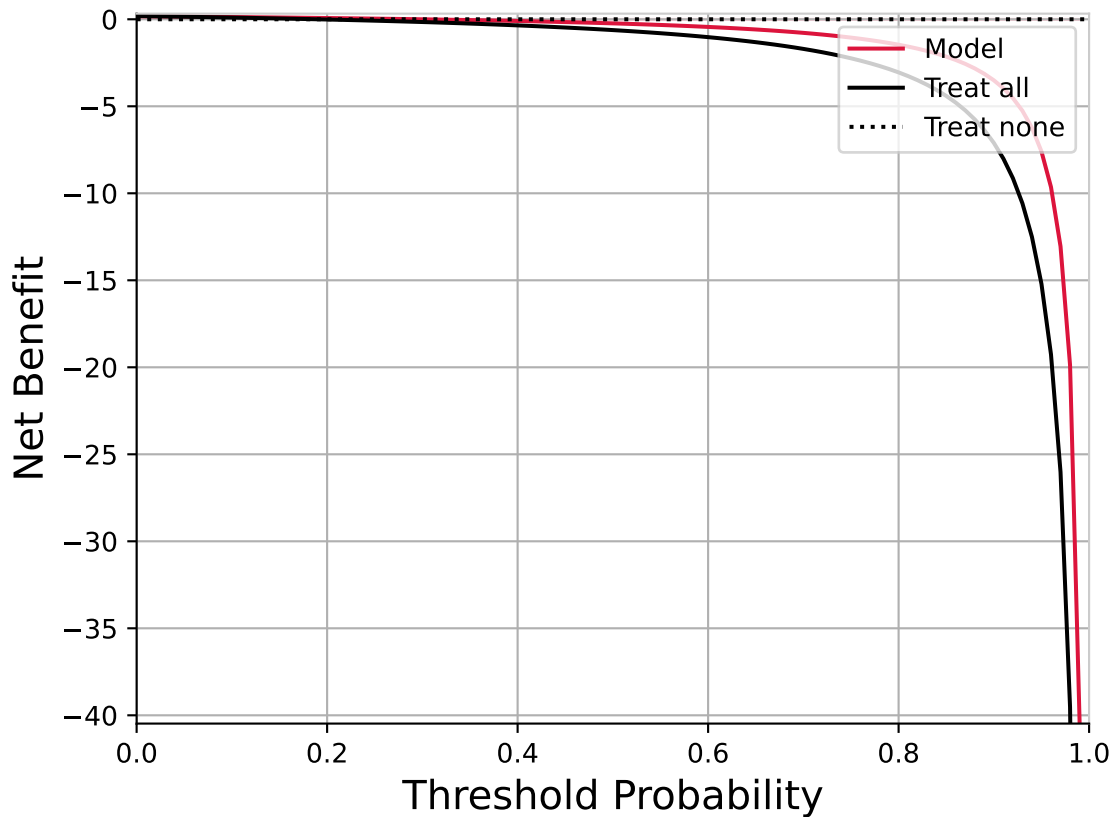

Supplement: Multimedia Appendix 2 [file medinform-v14-e75565-s002.zip › Naive Bayes_DCA validation.pdf]

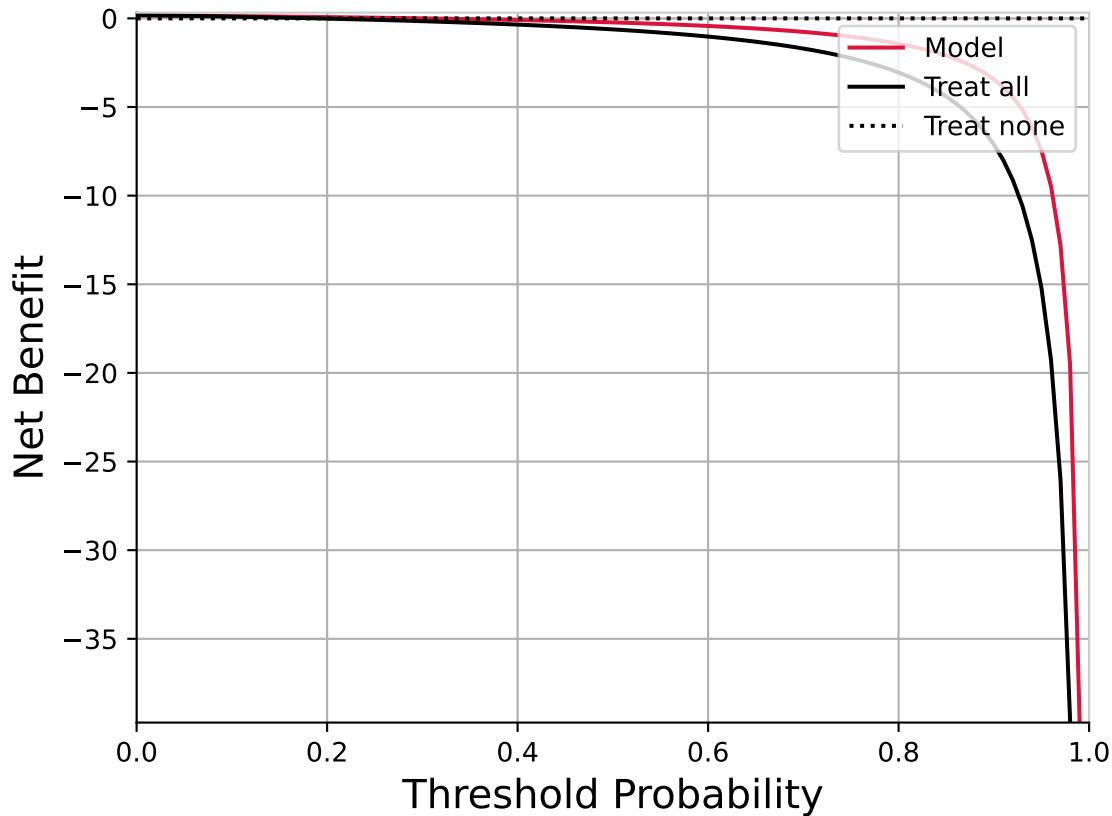

Supplement: Multimedia Appendix 2 [file medinform-v14-e75565-s002.zip › Random Forest_DCA validation.pdf]

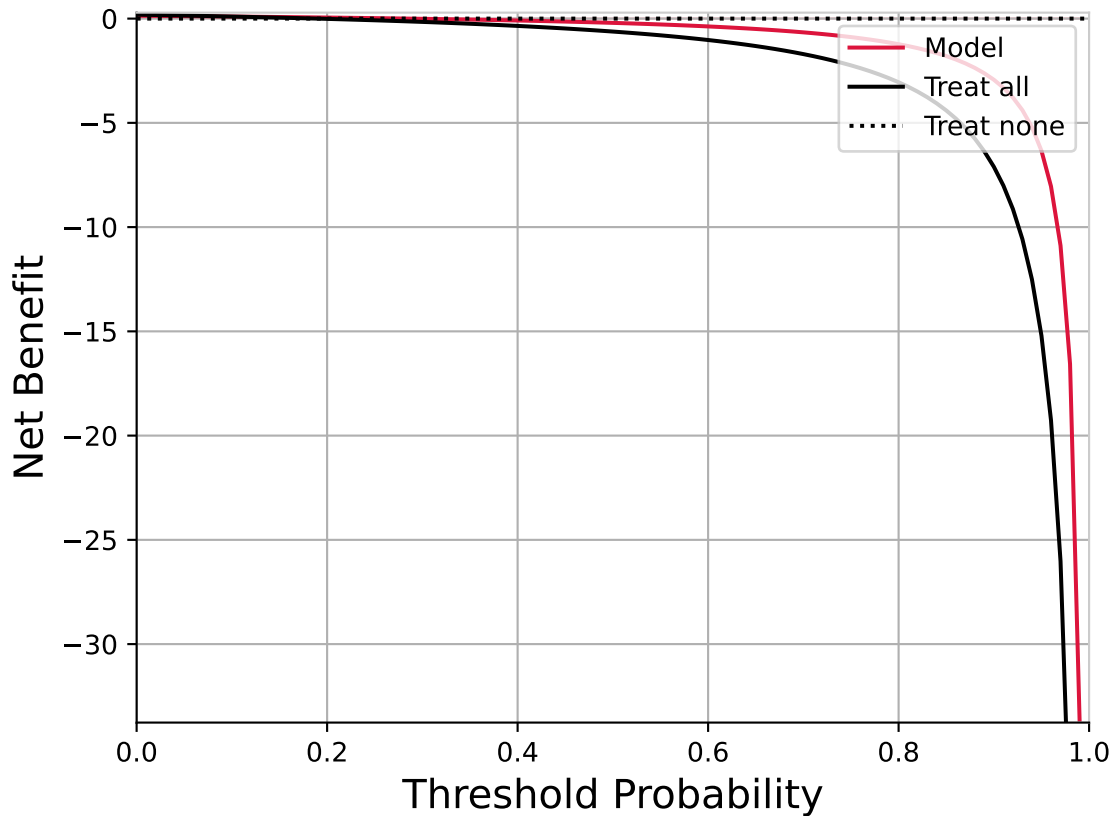

Supplement: Multimedia Appendix 2 [file medinform-v14-e75565-s002.zip › Ridge Regression_DCA validation.pdf]

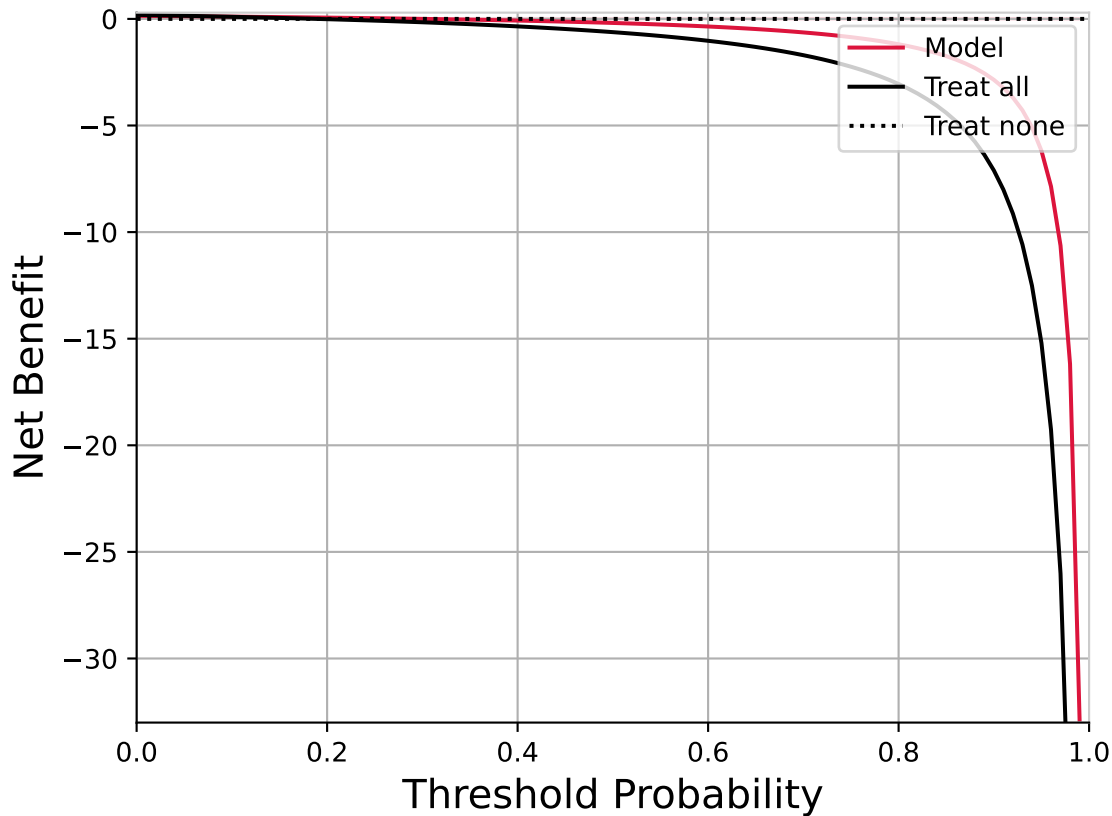

Supplement: Multimedia Appendix 2 [file medinform-v14-e75565-s002.zip › RidgeCV_DCA validation.pdf]

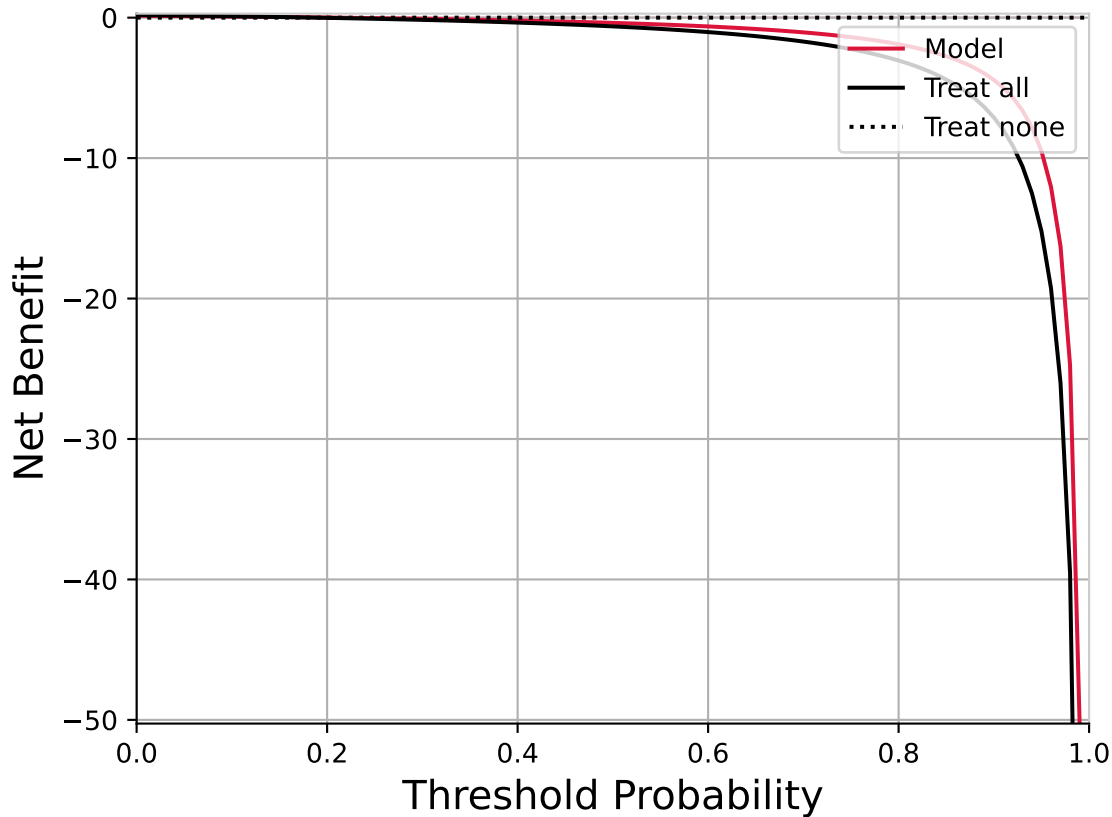

Supplement: Multimedia Appendix 2 [file medinform-v14-e75565-s002.zip › SGD_DCA validation.pdf]

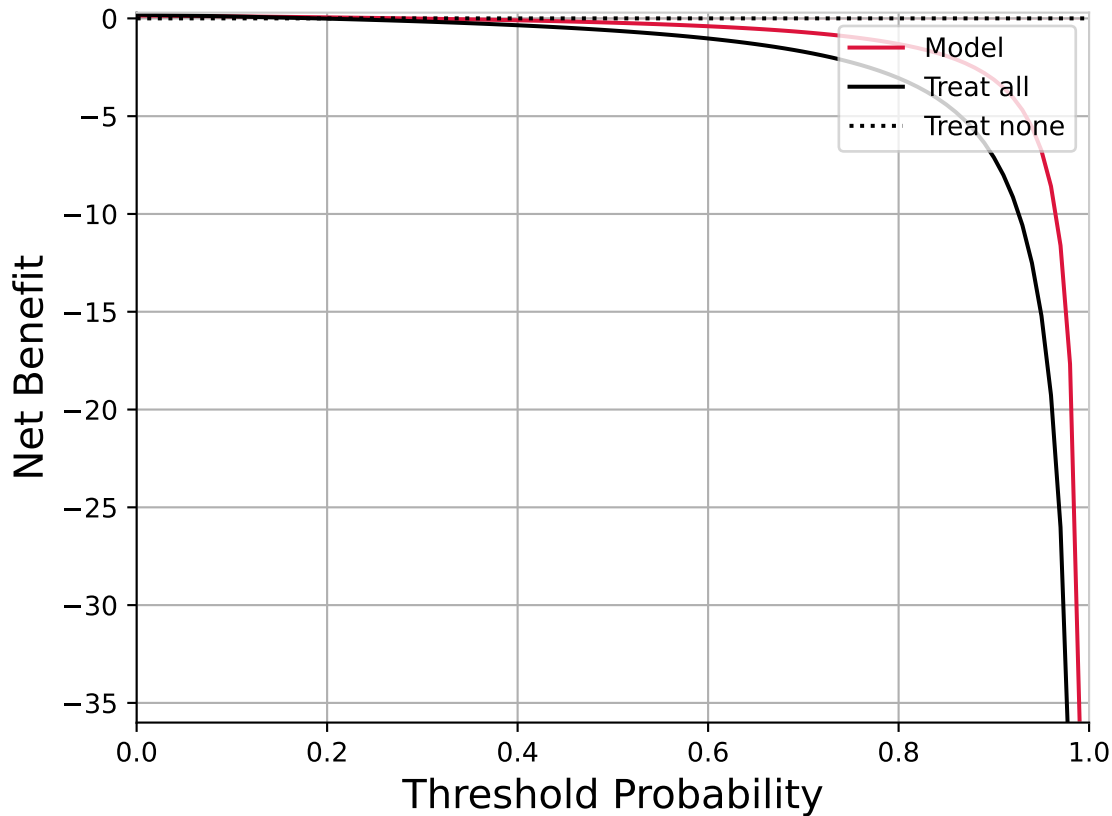

Supplement: Multimedia Appendix 2 [file medinform-v14-e75565-s002.zip › SVM_DCA validation.pdf]
